# Identification of a Methylation-Regulating Genes Prognostic Signature to Predict the Prognosis and Aid Immunotherapy of Clear Cell Renal Cell Carcinoma

**DOI:** 10.3389/fcell.2022.832803

**Published:** 2022-03-02

**Authors:** Li Zhang, Zhixiong Su, Fuyuan Hong, Lei Wang

**Affiliations:** ^1^ Department of Nephrology, Fujian Provincial Hospital, Shengli Clinical Medical College of Fujian Medical University, Fuzhou, China; ^2^ Department of Radiation Oncology, Fujian Cancer Hospital, Fujian Medical University Cancer Hospital, Fuzhou, China

**Keywords:** methylation, clear cell renal cell carcinoma, quantitative real-time transcription, immune checkpoint inhibitor, prognosis, risk signature

## Abstract

Methylation is one of the most extensive modifications of biological macromolecules and affects cell-fate determination, development, aging, and cancer. Several methylation modifications, including 5-methylcytosine and N6-methyladenosine, play an essential role in many cancers. However, little is known about the relationship between methylation and the prognosis of clear cell renal cell carcinoma (ccRCC). Here, we established a methylation-regulating genes prognostic signature (MRGPS) to predict the prognoses of ccRCC patients. We obtained ccRCC samples from The Cancer Genome Atlas and identified methylation-regulatingd genes (MRGs) from the Gene Set Enrichment Analysis database. We also determined differentially expressed genes (DEGs) and performed cluster analysis to identify candidate genes. Subsequently, we established and validated an MRGPS to predict the overall survival of ccRCC patients. This was also verified in 15 ccRCC samples collected from the Fujian Provincial Hospital via quantitative real-time transcription (qRT-PCR). While 95 MRGs were differentially expressed (DEGs1) between tumor and normal tissues, 17 MRGs were differentially expressed (DEGs2) between cluster 1 and 2. Notably, 13 genes common among DEGs1 and DEGs2 were identified as hub genes. In fact, we established three genes (*NOP2, NSUN6*, and *TET2*) to be an MRGPS based on their multivariate Cox regression analysis coefficients (*p* < 0.05). A receiver operating characteristic curve analysis confirmed this MRGPS to have a good prognostic performance. Moreover, the MRGPS was associated with characteristics of the tumor immune microenvironment and responses to inhibitor checkpoint inhibitors. Data from “IMvigor 210” demonstrated that patients with a low MRGPS would benefit more from atelozumab (*p* < 0.05). Furthermore, a multivariate analysis revealed that MRGPS was an independent risk factor associated with ccRCC prognosis (*p* < 0.05). Notably, a nomogram constructed by combining with clinical characteristics (age, grade, stage, and MRGPS risk score) to predict the overall survival of a ccRCC patient had a favorable predictive value. Eventually, our qRT-PCR results showed that tumor tissues had higher *NOP2* and *NSUN6* expression levels and lower *TET2* expression than normal tissues of ccRCC samples. While the proposed MRGPS comprising *NOP2*, *NSUN6*, and *TET2* can be an alternative prognostic biomarker for ccRCC patients, it is a promising index for personalized ICI treatments against ccRCC.

## Introduction

Clear cell renal cell carcinoma (ccRCC) is one of the most lethal malignancies of the genitourinary tract, accounting for 70–80% of renal cell carcinoma patients (Zhao et al., 2018). Despite substantial advances in the diagnosis and treatment of ccRCC, long-term prognosis remains far from satisfactory (Siegel et al., 2017). Approximately 20–30% of patients initially present with metastasis (Reiter et al., 2015), indicating that the current screening index for ccRCC is inadequate; thus, it is necessary to immediately identify an aggressive diagnostic marker for ccRCC. In addition, approximately 30–40% of patients with localized ccRCC relapse or exhibit metastasis within 2 years of undergoing radical surgeries (Miao et al., 2018). This implies that the ccRCC patient population is greatly heterogeneous and highlights the inaccuracies in the existing staging system integrated with clinicopathological characteristics.

Interestingly, ccRCC is a highly immunogenic tumor characterized by an abundance of suppressed immune cells (Díaz-Montero et al., 2020). A randomized phase II study has demonstrated that immune checkpoint inhibitor (ICI) monotherapy exhibits non-inferiority efficacy to sunitinib (Mcdermott et al., 2018). However, a CheckMate-214 trial (Cella et al., 2019; Albiges et al., 2020) has revealed that nivolumab combined with iplimumab has positive outcomes compared with sunitinib. Thus, this combination has been approved by the United States Food and Drug Administration as a frontline therapeutic approach for ccRCC patients with intermediate severity. Nonetheless, the objective response rates (ORRs) of avelumab, pembrolizumab, and nivolumab are 16, 36, and 17%, respectively (Tzeng et al., 2021), whereas that of avelumab combined with nivolumab is 42% (Cella et al., 2019). Additionally, continuing treatment with nivolumab has been found to be associated with reduced tumor burden in approximately 50% of patients (Hellmann et al., 2018). Hence, an aggressive biomarker, except PD-1/PD-L1, tumor mutation burden (TMB), and microsatellite status, is urgently warranted in ICI management for ccRCC.

Methylation is one of the most abundant modifications that is widespread across all biological processes. It involves an alkylation reaction, wherein a methyl group replaces a hydrogen atom (Michalak et al., 2019). Methyltransferases, also called “writers,” use the methyl donor S-adenosylmethionine to catalyze methylation; “writers” cooperate with dedicated “erasers” (demethylases) and methyl “readers” (Dawson and Kouzarides, 2012). Genomic studies have demonstrated that hypo- and/or hyper-methylation occur in various enzymes and can result in loss of histone modification (Michalak et al., 2019). Few examples include mutations in metabolic enzymes that regulate histone and DNA demethylation and somatic mutations in core histone genes (You and Jones, 2012). In fact, previous studies have demonstrated that aberrant changes in DNA or RNA methylation can be prospectively utilized in the diagnosis, prognosis, and individualized treatment of various cancers, including ccRCC (Fang et al., 2020; Zhang et al., 2020; Li et al., 2021). Therefore, we systematically analyzed the transcriptomic data of ccRCC patient tissues to identify methylation-regulating genes (MRGs) and accurately predict the prognoses and guide the ICI management of ccRCC patients.

## Materials and Methods

### Patients and Datasets

We retrieved 359 human MRGs from the Gene Set Enrichment Analysis (GSEA) database (https://www.gsea-msigdb.org/gsea/index.Jsp; Supplementary Table S1) (Subramanian et al., 2005). Moreover, we obtained RNA sequencing (RNA-Seq) expression profile dataset of 537 ccRCC patients and 72 corresponsonding normal samples from The Cancer Genome Atlas (TCGA; https://portal.gdc.cancer.gov/) (Tomczak et al., 2015). The clinicopathological characteristics and survival data of these patients was also retrieved from TCGA. The RNA-seq profiles and clinical data of“IMvigor 210” cohort were obtained from http://research-pub.gene.com/IMvigor210CoreBiologies/.

Furthermore, 15 frozen, surgically resected tumor specimens were acquired from patients pathologically diagnosed with ccRCC at the Fujian Provincial Hospital (FPH) between December 2018 and December 2020. Additionally, we validated the immunohistochemical staining of prognostic genes using The Human Protein Atlas (HPA) database (http://www.proteinatlas.org/) (Uhlén et al., 2015). This study was approved by the ethics committee of the FPH.

### Identification of Methylation-Regulating Hub Genes

Based on the RNA-seq data of the ccRCC samples (537 tumors vs 72 normal samples) obtained from TCGA, we analyzed the differentially expressed genes (DEGs1) between tumor and normal tissues. We also functionally explored the biological properties of MRGs in the TCGA ccRCC patients by clustering ccRCC patients into different clusters using the “ConsensusClusterPluspackage” (Wilkerson and Hayes, 2010) (http://www.bioconductor.org/; 1,000 iterations and resampling rate of 80%). The cumulative distribution function (CDF) and delta area were considered to determine the optimal number of groups (k). Subsequently, we identified DEGs between the different clusters (DEGs2) and defined the hub genes as genes common to both DEGs1 and DEGs2.

### Construction and Validation of Methylation-Regulating Genes Prognostic Signature

We divided TCGA patients into training and validation cohorts at a ratio of 3:7 (11 samples were deleted because their OS was 0 or unknown), and prognostically significant hub genes (*p* < 0.05) were screened by univariate Cox regression analysis. In fact, these candidate genes were used to establish a methylation-regulating genes prognostic signature (MRGPS) via multivariate Cox regression analysis. The risk score for each patient was determined using the following formula:

$$\mathrm{Risk}\;\mathrm{score}=\sum_{\mathrm{i=1}}^{n}\mathrm{Coef}\left(i\right)\times x\left(i\right)$$

Thereafter, the patients were classified into low-risk and high-risk groups based on the median risk score. We determined the prognostic ability of the MRGPS in the training cohort by generating Kaplan–Meier survival curves and receiving operating characteristic (ROC) curves using the R packages “survminer” and “survivalROC”. The prognostic performance of this MRGPS was further tested in the testing cohort in the same manner as mentioned above.

### Functional Analysis

We conducted Gene Ontology (GO) and Kyoto Encyclopedia of Genes and Genomes (KEGG) analysis to analyze the main function using the “clusterProfiler" (Yu et al., 2012) R package and visualized it using the “Treemap” (Liu et al., 2021) and “ggplot2” packages. Additionally, GSEA was performed to understand the biological processes prevalent in the different subgroups using the “clusterProfiler” R package. Predefined gene sets were identified in the GSEA using the GO Biological Process; 5,000 permutations were performed to determine the *p* values of these gene sets. Significant pathways were defined as having a *p* value of <0.05 and a false discovery rate (FDR) of <0.05 (Powers et al., 2018).

### Immune Score and Immunotherapy Benefits Analyses

We conducted a single sample GSEA (ssGSEA) analysis, where (Bustin and Mueller, 2005)in we analyzed 20 immune cells of 537 ccRCC samples based on the expression profile of a single sample; we used the “gsva” R package to perform this analysis (Hänzelmann et al., 2013). The ESTIMATE algorithm (i.e., the “estimate” R package) was used to calculate the immune score of each patient. Subsequently, we assessed the immune score difference between the two cluster subgroups. A semi-quantitative analysis of 22 immune cell types in the two MRGPS groups was performed using CIBERSORT via the “cibersort” R package (Chen et al., 2018). Moreover, we calculated tumor immune dysfunction and exclusion (TIDE) and microsatellite instability (MSI) scores from the website of http://tide.dfci.harvar.edu to assess the potential efficacy of ICIs in the two MRGPS subgroups (Fu et al., 2020). We also compared the somatic mutations between the two MRGPS subgroups by obtaining the TMB, i.e., the total number of somatic mutations.

### Predicting the Benefits of MRGPS for Tyrosine Kinase Inhibitors

Since VEGFR-targeted therapy remains the first line of treatment for ccRCC, we explored the sensitivity of TKIs, such as sunitinib, sorafenib, pazopanib, and axitinib, stratified by MRGPS. The sensitivity of each TKI was evaluated by IC_50_ calculation using the “pRRophetic” package (Geeleher et al., 2014), and the corresponding data were obtained from the Genomics of Drug Sensitivity in Cancer database (Yang et al., 2013).

### Development of Risk Prediction Model

Furthermore, we conducted a multivariate Cox analysis to evaluate whether the signature-based risk score was independent of other clinical characteristics. The testing cohort was used to further test the performance of the signature in the same manner mentioned above. Thereafter, we generated a nomogram consisting of the current MRGPS and clinical characteristics with *p* < 0.1. This helped predict the 1-, 3- and 5-years overall survival (OS) of the TCGA ccRCC patients using the “rms” package. Additionally, we evaluated this nomogram using the calibration curve, ROC curve, and decision-making curve (DCA).

### Quantitative Reverse Transcription PCR

Relative quantitation of the 15 paired mRNAs was determined by quantitative reverse transcription polymerase chain reaction (qRT-PCR; SuperScript IV Reverse Transcriptase 18090010; Thermo Fisher, United States). The amplification reactions were performed as described previously (Bustin and Mueller, 2005). NSUN6-specific primers were: forward primer, 5′-ATC​TGC​GTC​CGT​TTC​ACC-3′ and reverse primer, 5′-GCT​TCC​ACC​ACA​CCT​CAT​C-3'. NOP2-specific primers were: forward primer, 5′-GGG​CAC​AGA​CAC​ACA​AAC​A-3′ and reverse primer, 5′-GAA​CGG​ATG​GGA​GAC​ACA​G-3'. TET2-specific primers were: forward primer, 5′-CAC​AAC​CAT​CCC​AGA​GTT​CA-3′ and reverse primer, 5′-ACT​TCC​TCC​AGT​CCC​ATT​TG-3'. Human β-actin-specific primers were: forward primer, 5′-GAA​GAG​CTA​CGA​GCT​GCC​TGA-3′ and reverse primer 5′- CAG​ACA​GCA​CTG​TGT​TGG​CG-3'. Data analysis was performed using the ΔΔCT method.

### Statistical Analyses

Distributed data were compared by performing the Student’s t-test and Wilcoxon test, whereas proportion differences were calculated by the chi-square test. Additionally, component analysis in subgroups were compared by the Fisher’s test. While survival differences between different groups were assessed via the log-rank test, prognostic factors were identified by the Cox regression analyses. All statistical analyses were performed using RStudio version 4.0.3, and two-sided *p* < 0.05 was considered as statistically significant.

## Results

### Identification of Methylation-Regulating Hub Genes

**FIGURE 1:**
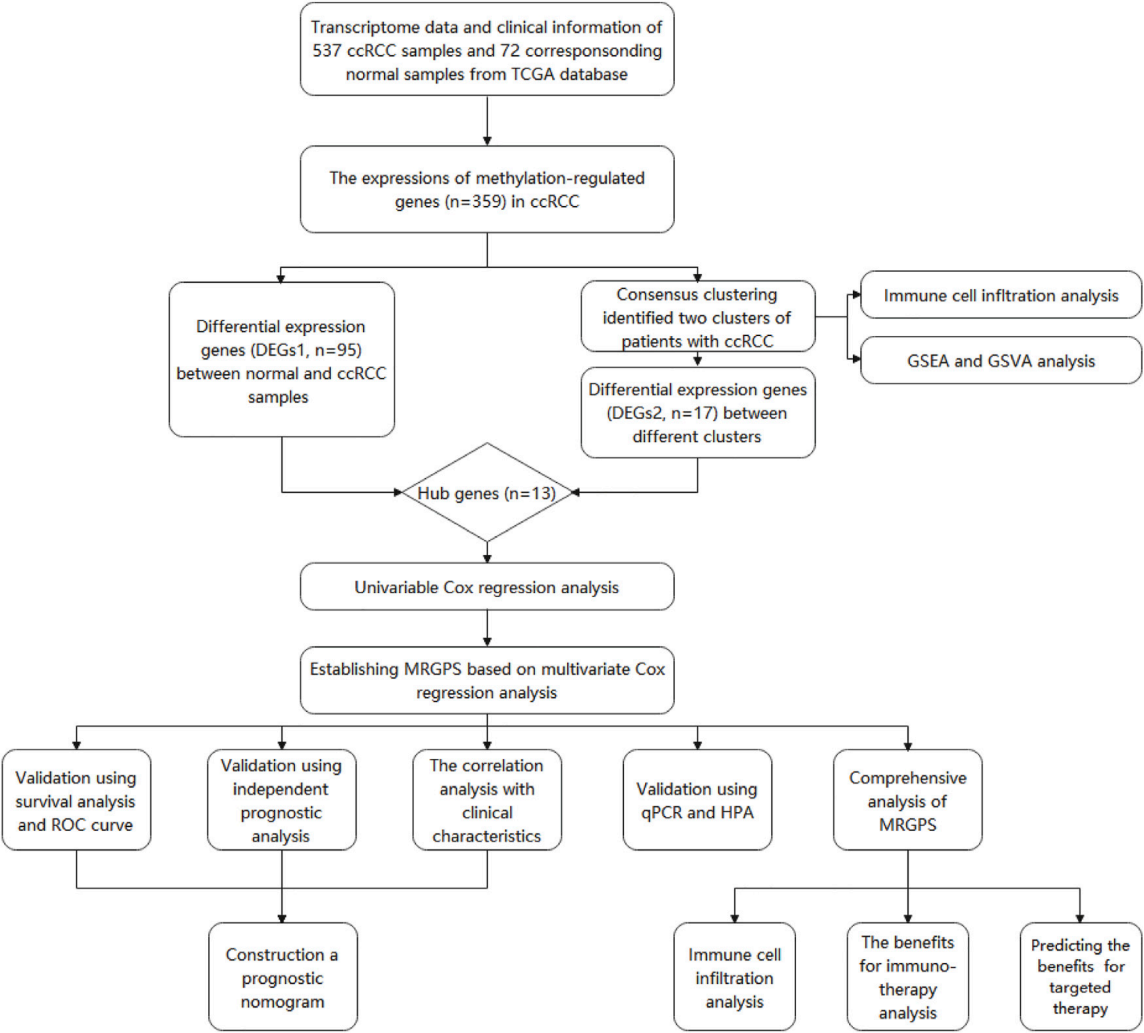
The entire analytical process of the study.

**FIGURE 2:**
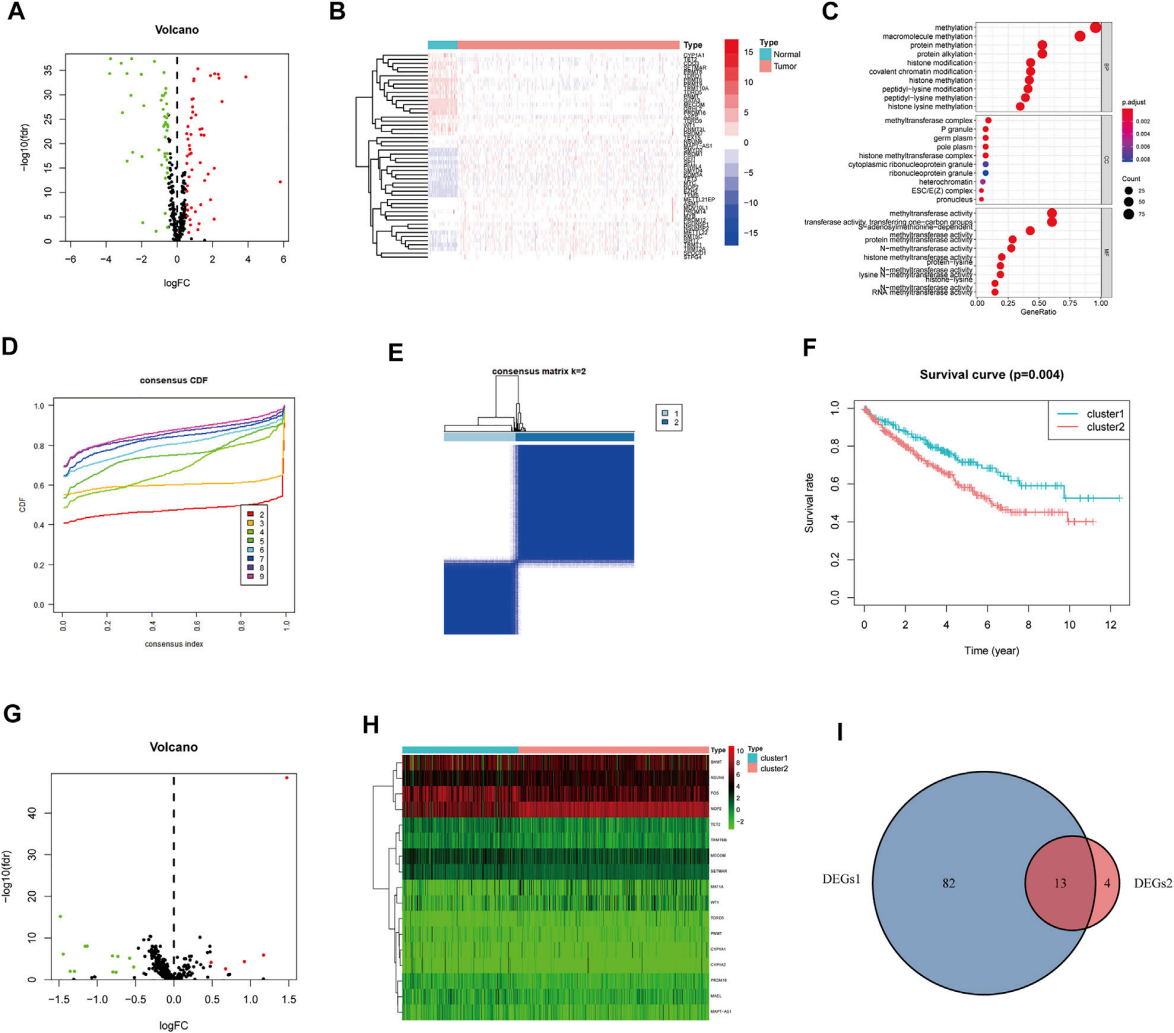
Identification of methylation-regulating Hub Genes. **(A)** Volcano plot demonstrates DEGs1. **(B**) Heatmap demonstrates the top 50 DEGs1. **(C)** The Gene Ontology (GO) analysis of DEGs1 in ccRCC. **(D)** Consensus clustering cumulative distribution function (CDF) for k = 2 to 9. **(E)** The consensus heatmap showed the ccRCC patients was divided into two distinct clusters when k = 2. **(F)** Overall Survival (OS) analysis of different clusters in the TCGA dataset. **(G)** Volcano plot demonstrates the DEGs2. **(H)** Heatmap demonstrates the DEGs2. **(I)** Venn diagram demonstrates the intersect between DEGs1 and DEGs2.

The entire analytical process of this study is presented in Figure 1, and the clinical characteristics of the ccRCC patients in the TCGA and FPH cohorts are listed in Table 1. Additionally, Figure 2A presents all 95 DEGs among tumor and normal tissues (DEGs1: FDR<0.05 and |log2FC|>0.5), including 51 upregulated and 44 downregulated genes; the top 50 DEGs are presented in a heatmap (Figure 2B). The GO analysis of DEGs1 revealed that methylation-relate biological process (BP), cellular component (CC), and molecular function (MF) were enriched in tumor tissues (Figure 2C).

**TABLE 1:** Clinical characteristics of the ccRCC patients in TCGA cohort and FPH cohort.

Characteristic	TCGA cohort	FPH cohort
n (%)	537 (100%)	15 (100%)
Age, n (%)	—	—
≤65	352 (65.55%)	11 (73.33%)
>65	185 (34.45%)	4 (26.67%)
Gender, n (%)	—	—
Female	191 (35.57%)	6 (40.00%)
Male	346 (64.43%)	9 (60.00%)
Histologic grade, n (%)	—	—
G1	14 (2.61%)	NA
G2	230 (42.83%)	NA
G3	207 (38.55%)	NA
G4	78 (14.53%)	NA
NA	8 (1.48%)	NA
Pathologic stage, n (%)	—	—
Stage I	269 (50.09%)	12 (80.00%)
Stage II	57 (10.61%)	2 (13.33%)
Stage III	125 (23.28%)	1 (4.67%)
Stage IV	83 (15.46%)	0 (0%)
NA	3 (0.56%)	0 (0%)
T stage, n (%)	—	—
T1	275 (51.21%)	12 (80.00%)
T2	69 (12.85%)	2 (13.33%)
T3	182 (33.89%)	1 (4.67%)
T4	11 (2.05%)	0 (0%)
N stage, n (%)	—	—
N0	240 (44.69%)	15 (100%)
N1	17 (3.17%)	0 (0%)
NA	280 (52.14%)	0 (0%)
M stage, n (%)	—	—
M0	426 (79.33%)	15 (100%)
M1	79 (14.71%)	0 (0%)
NA	32 (5.96%)	0 (0%)

Subsequently, we performed consensus clustering to explore the molecular characteristics between different MRG expression samples. We observed a relative change in the CDF of the consensus cluster from k = 2 to k = 9 (Figure 2D); the delta area under the CDF curve from k = 2 to 9 is depicted in Supplementary Figure S1H. The corresponding heatmap presents the results of this consensus from k = 2 to 9 (k = 2, Figure 2E; k = 3–9, Supplementary Figure S1A–G). The criteria for deciding the cluster number was determined by a relatively high consistency and a low variation coefficient and an appreciable increase in the area under the CDF curve. Thus, after comprehensive consideration, we chose k = 2 as the optimal cut-off for the clusters number.

A significant difference in the OS was observed between patients of clusters 1 and 2 (*p* < 0.001, Figure 2F). To determine which genes contributed to this difference in prognosis, we first identified 17 genes as DEGs between cluster 1 and cluster 2 (DEGs2: FDR<0.05, |log2FC|>0.5; Figure 2G); these genes are also represented in a heatmap (Figure 2H). Eventually, 13 overlapping genes between DEGs1 and DEGs2 were identified as the hub genes (Figure 2I, Supplementary Table S2).

**FIGURE 3:**
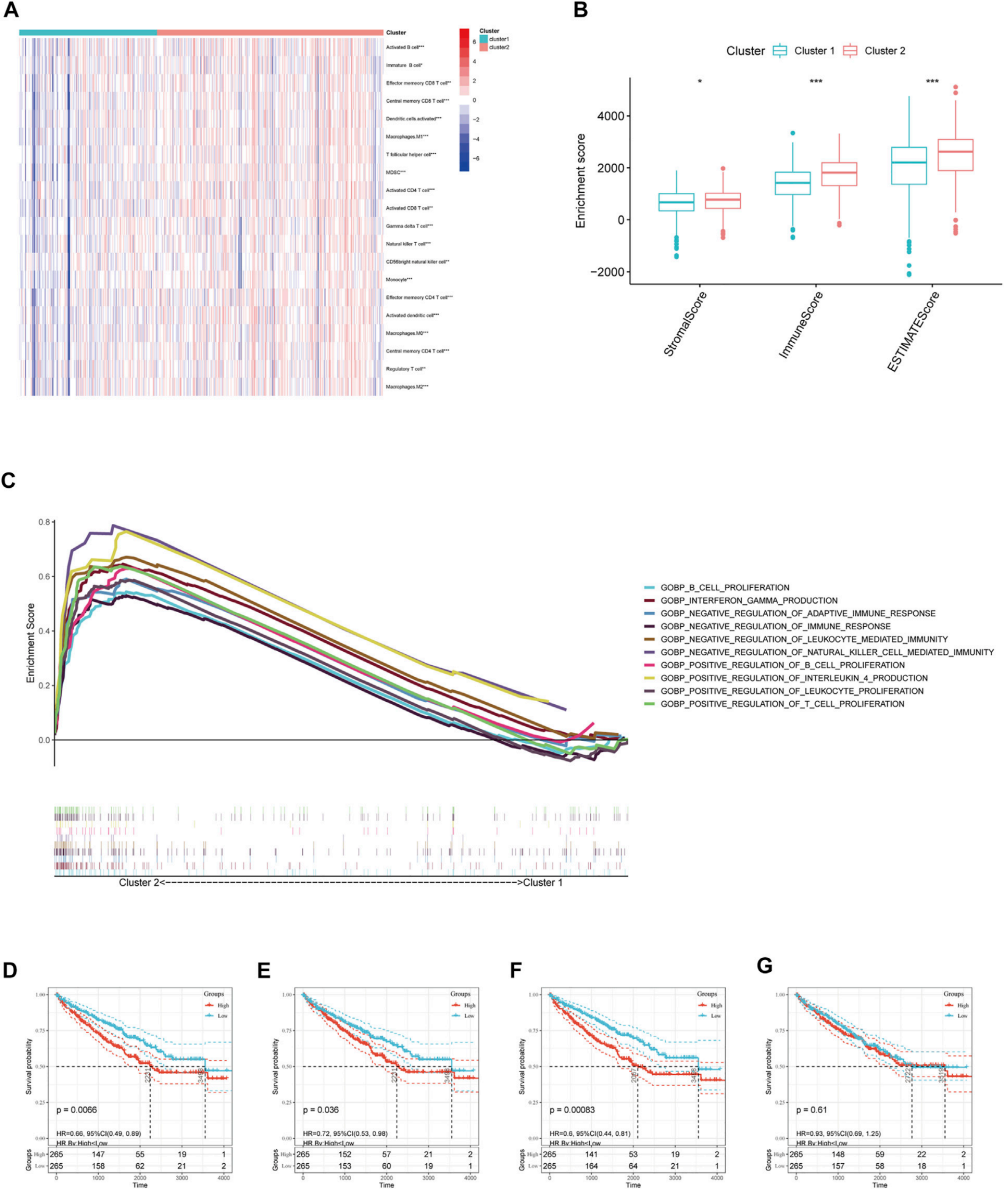
Immune cell infiltration analysis and GSEA analysis between different clusters. **(A)** Estimated abundance of 20 immune cells using ssGSEA. **(B)** Tumor microenvironment (TME) estimate score in different clusters. **(C)** GSEA delineation of the biological pathways which enrich in cluster 2 using the gene set “c5. go.bp.v7.4. symbols”. Overall Survival (OS) analysis in different GSVA score of **(D)** negative regulation of adaptive immune response, **(E)** negative regulation of immune response, and **(F)** negative regulation of leukocyte-mediated immunity, **(G)** negative regulation of natural killer cell-mediated immunity in TCGA-ccRCC patients. Significant statistical differences between the two clusters were assessed using the Wilcoxon test (ns, *p* > 0.05; *, *p* < 0.05; **, *p* < 0.01; ***, *p* < 0.001).

We further evaluated the molecular characteristics of the different clusters by conducting immune-related analyses between clusters 1 and 2. The ssGSEA demonstrated that cluster 2 had a high abundance of approximately all immune cell types compared to cluster 1 (*p* < 0.05, Figure 3A). Moreover, the tumor microenvironment estimate scores, including the stromal, immune, and total scores, were higher in cluster 2 than those in cluster 1 (*p* < 0.05, Figure 3B). Notably, immune-related signaling pathways were enriched in cluster 2, as determined by the GSEA (Figure 3C and Supplementary Table S3). Furthermore, we obtained the enrichment score of each negative immune-related signaling pathway in each ccRCC sample by performing a gene set variation analysis (GSVA). Consequently, we observed significant survival differences between high- and low-GSVA scores regarding the negative regulation of adaptive immune response, negative regulation of immune response, and negative regulation of leukocyte-mediated immunity (*p* < 0.05, Figures 3D–F); however, this did not hold true for the negative regulation of natural killer cell-mediated immunity (*p* > 0.05, Figure 3G).

### Construction and Validation of the Methylation-Regulating Genes Prognostic Signature

**FIGURE 4:**
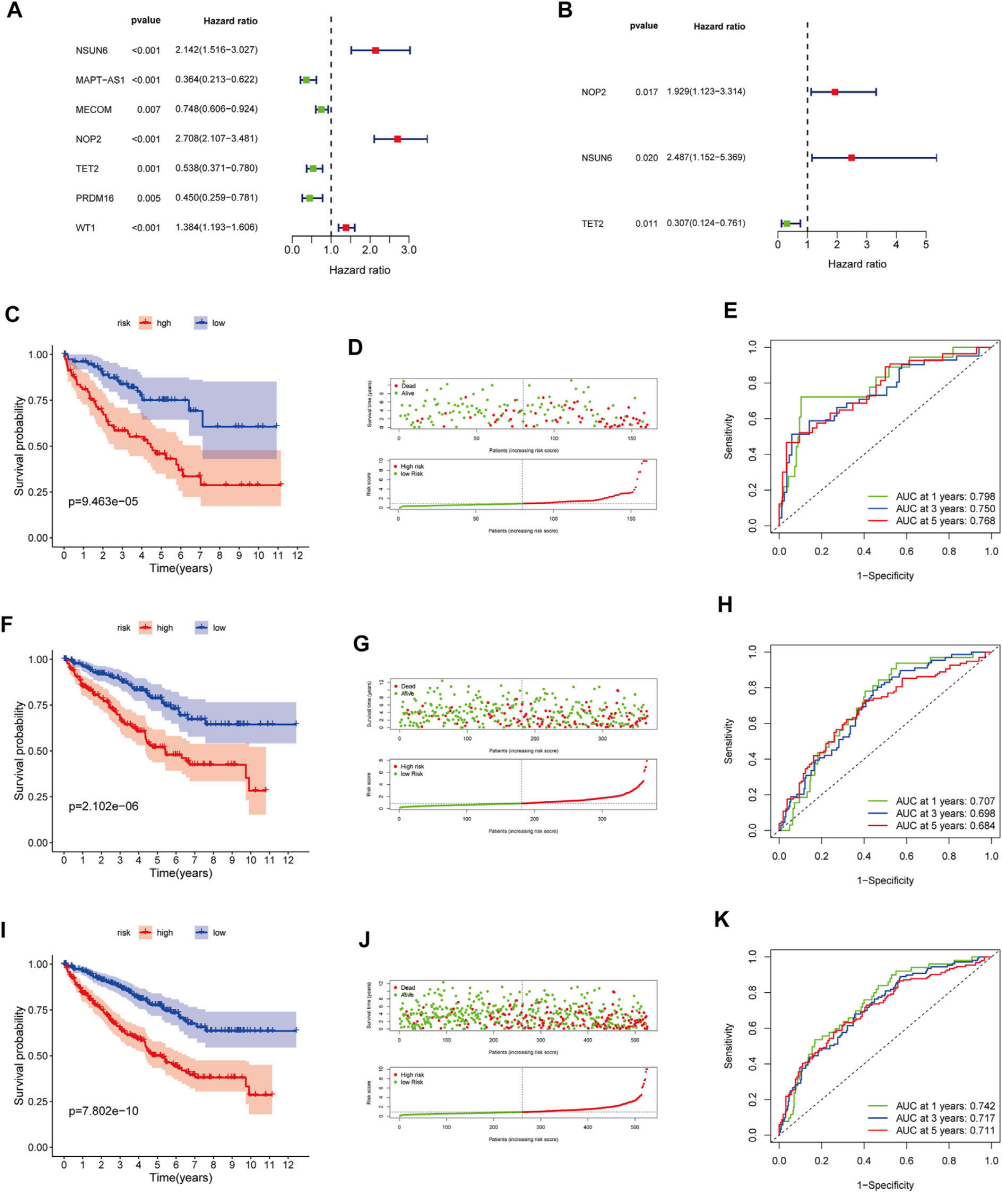
Construction and validation of the MRGPS. **(A)** Forrest plot of the univariate Cox regression analysis in the training cohort. **(B)** Forrest plot of the multivariate Cox regression analysis in the training cohort. Kaplan-Meier analysis, risk score analysis and ROC curve of the MRGPS inthe training cohort **(C–E)**, validation cohort **(F–H)**, and whole TCGA cohort **(I–K)**.

In the training cohort, we screened prognosis-associated seven hub genes by univariate Cox regression analysis (Figure 4A). Then, a multivariate Cox regression analysis was conducted to screen the optimal model and was depicted in Figure 4B; based on their regression coefficients, three MRGS (*NOP2, NSUN6,* and *TET2*) were identified to form an MRGPS. The MRGPS score of each patient was calculated according to the following formula: *Risk score =* [NOP2 expression*(*0.656940513*)] *+* [NSUN6 expression*(0.911107243)] + [TET2 expression*(-1.180533124)]. Considering the median score as the cut-off value, patients in the training cohort were divided into low- and high-risk groups; these patients had apparent survival differences (*p* < 0.001, Figure 4C). The corresponding risk scores and survival statuses are presented in Figure 4D. The ROC curves demonstrated the excellent predictive capability of the current MRGPS with 1-, 3-, and 5-years AUCs being 0.798, 0.750, and 0.768, respectively (Figure 4E). Likewise, the advantages of the current MRGPS were observed in the validation (Figures 4F–H) and the whole cohorts (Figures 4I,J).

In addition, relationships between clinicopathological characteristics and risk scores were further explored. As shown in Supplementary Figure S2A, differences were observed regarding the age (age ≤65 years, age >65 years), differentiation (G1, G2, G3, G4), T stage (T1, T2, T3, T4), M stage (M0, M1), and cancer stage (I, II, III, IV). Furthermore, Kaplan–Meier survival curves showed that the high-risk patients had worse prognoses than the low-risk patients in the following attributes: age ≤65 years, age >65 years, male sex, female sex, G1-2, G3-4, T1-2, T3-4, M0, M1, stage I–II, and stage III–IV (*p* < 0.05, Supplementary Figure S2B–M).

### Immune Analyses and Immunotherapy

**FIGURE 5:**
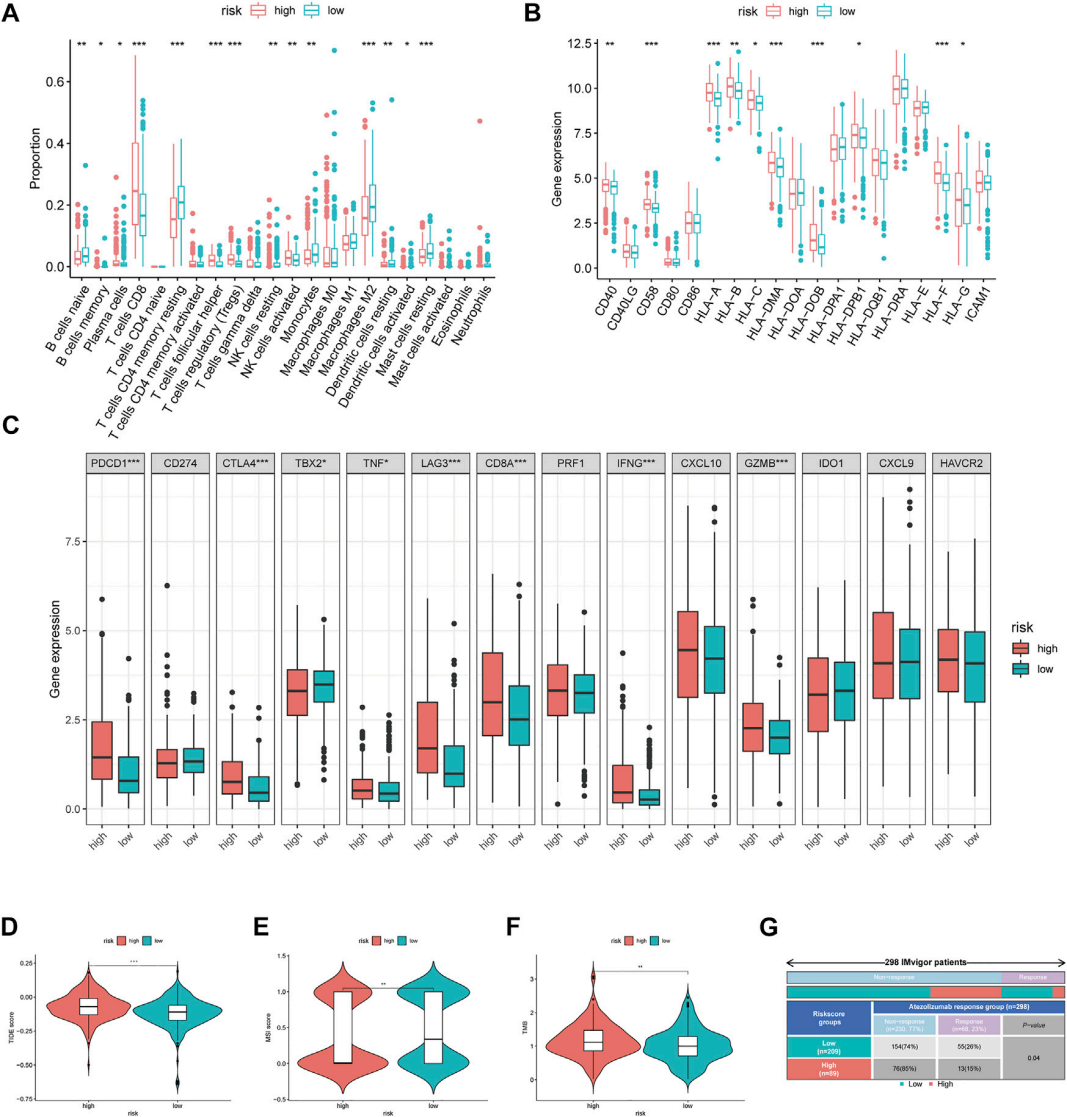
Immune-related analysis between different MRGPS subgroup. **(A)** The proportions of TME cells in different MRGPS subgroups. **(B)** Relative expression of MHC molecules, co-stimulatory molecules, and adhesion factors. **(C)** Association of MRGPS with immune checkpoint molecules. **(D)** TIDE, **(E)** MSI, **(F)** TMB score in different MRGPS subgroups. **(G)** Distribution of immune response to ICIs therapy in different MRGPS subgroups in IMvigor patients. Significant statistical differences between the two subgroups were assessed using the Wilcoxon test (ns, *p* > 0.05; *, *p* < 0.05; **, *p* < 0.01; ***, *p* < 0.001).

We further explored the immune microenvironment characteristics of patients belonging to the different risk subgroups by conducting immune cell infiltration and immune function analysis on the TCGA cohort patients. We observed significantly decreased number of naive B cells, memory B cells, plasma cells, CD4+T cells, CD4+T memory cells, gamma T cells, resting NK cells, M0/M1/M2 and resting dendritic cells, activated dendritic cells, and resting mast cells and also observed increased number of CD8+T cells and regulatory T cells in the high-risk group (*p* < 0.05, Figure 5A). Relative expression levels of MHC molecules and co-stimulatory molecules and adhesion factors, such as CD40, CD58, HLA-A, HLA-B, HLA-C, HLA-DMA, HLA-DOB, HLA-DPB1, and HLA-F, were all higher in the high-risk group than those in the low-risk group (*p* < 0.05, Figure 5B). Importantly, the expression levels of immune checkpoint proteins, such as PDCD1, CTLA4, TBX2, TNF, LAG3, CD8A, IFNG, and GZMB were all significantly higher in the high-risk group than those in the low-risk group (*p* < 0.05, Figure 5C).

Furthermore, patients in the high-risk group were found to have a higher TIDE score, lower MSI score, and higher TMB than those in the low-risk group (*p* < 0.05, Figures 5D–F). This suggested that low-risk patients may benefit more from immunotherapy compared to high-risk ones according to the current MRGPS. We further validated this observation using the “IMvigor 210” dataset containing clinical information and RNA-seq data of metastatic urothelial cancer patients who were treated with the ICI atezolizumab (PD-L1 inhibitor). Remarkably, data from the 298 IMvigor patients also validated the clinical utility of the current MRGPS in response to atezolizumab (*p* < 0.05, Figure 5G).

### Potential Biological Pathway Analysis of Methylation-Regulating Genes Prognostic Signature

**FIGURE 6:**
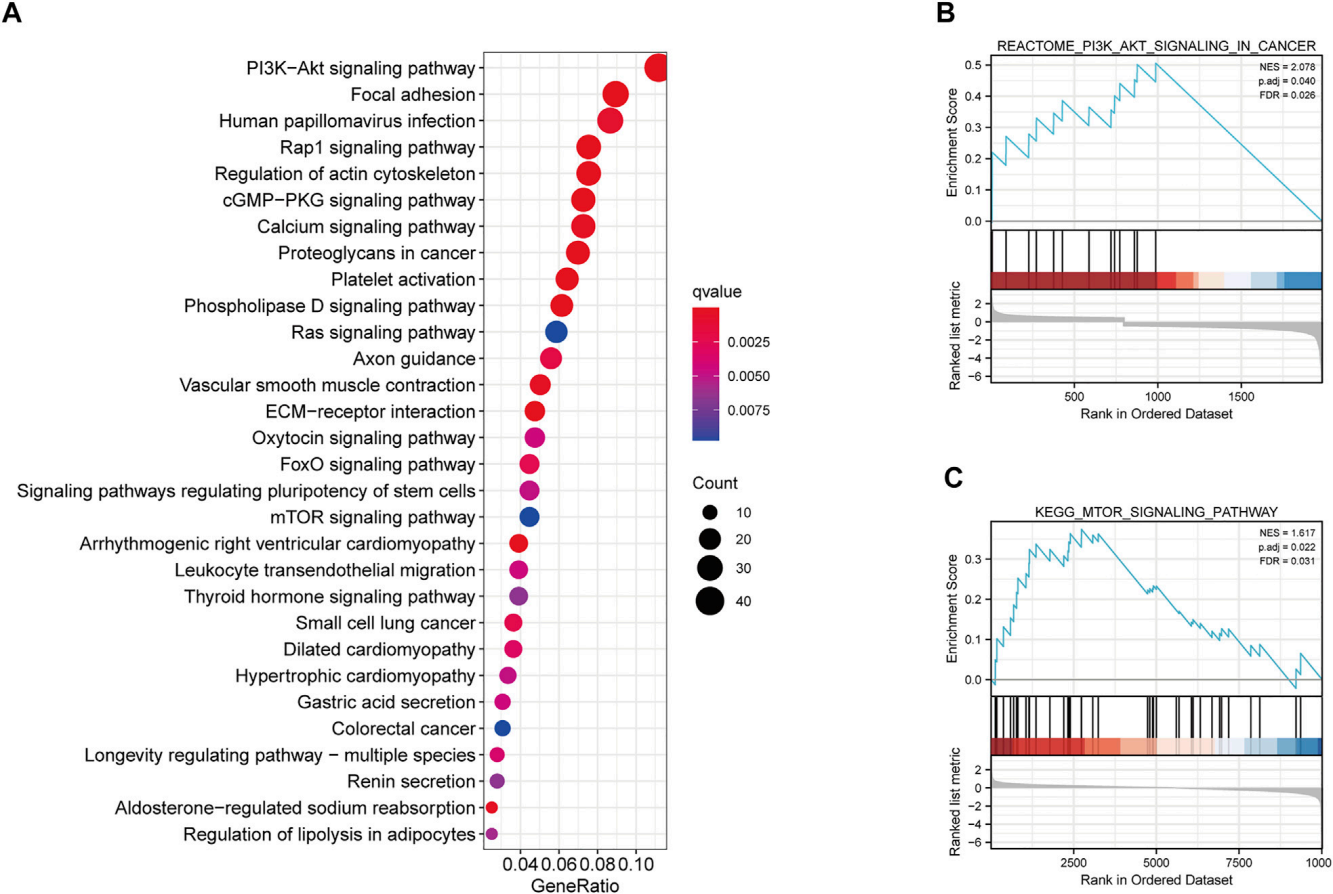
KEGG and GSEA analysis of MRGPS. **(A)** KEGG analysis of the DEGs between high- and low-risk groups. **(B)** PI3K−Akt signaling pathway and **(C)** mTOR signaling pathway were identified in the high-risk group.

We further determined the potential biological pathways prevalent in different risk group by performing a KEGG analysis on the DEGs among high- and low-risk groups (FDR<0.05 and |log2FC|>0.5). We observed that the “PI3K−Akt signaling pathway”, “mTOR signaling pathway”, “Ras signaling pathway” and other carcinogenesis-related pathways were enriched in the high-risk group (Figure 6A). Furthermore, the GSEA revealed that the high-risk group had higher enrichment score for the PI3K-AKT and mTOR pathways compared to the low-risk group (Figures 6B,C). These results revealed that the MRGPS possibly promotes cancer development by activating these pathways.

### VEGF Family Expressions and TKI Sensitivity

**FIGURE 7:**
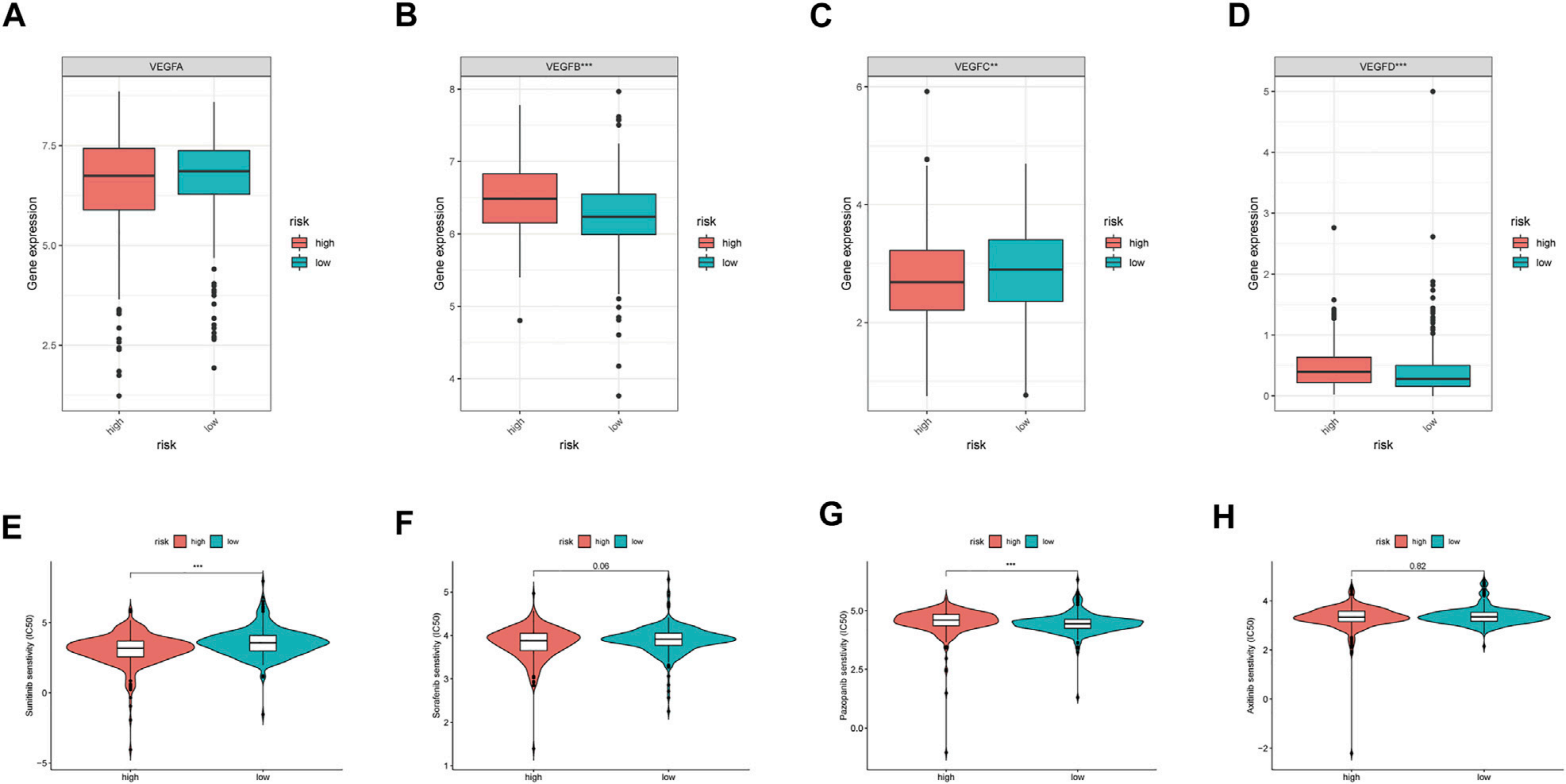
VEGF family expressions and the sensibility of TKI inhibitors in different MRGPS subgroups. **(A–D)** VEGF family expressions in different MRGPS subgroups. Drug susceptibility analysis between different MRGPS subgroups about **(E)** Sunitinib, **(F)** Sorafenib, **(G)** Pazopanib, **(H)** Axitinib. Wilcoxon test (ns, *p* > 0.05; *, *p* < 0.05; **, *p* < 0.01; ***, *p* < 0.001).

As the VEGF family was an important molecular target, we compared their expression levels in high- and low-risk groups. Consequently, no significant differences in VEGFA expression were observed between the two groups (*p* > 0.05, Figure 7A). However, VEGFB and VEGFD expression levels were significantly upregulated in the high-risk group (*p* < 0.05, Figures 7B,D), whereas that of VEGFC was significantly downregulated in the high-risk group (*p* < 0.05, Figure 7C). Further analysis revealed that sunitinib had lower IC_50_ the higher-risk group than that in the low-risk groups (*p* < 0.05, Figure 7E). In contrast, pazopanib (*p* < 0.05, Figure 7G), but not sorafenib and axitinib (both *p* > 0.05, Figures 7F,H), had lower IC_50_ in the low-risk group than that in the high-risk group. These results indicated that different risk groups had varying susceptibilities for different targeted drugs.

### Methylation-Regulating Genes Prognostic Signature-Based Nomogram Construction

**FIGURE 8:**
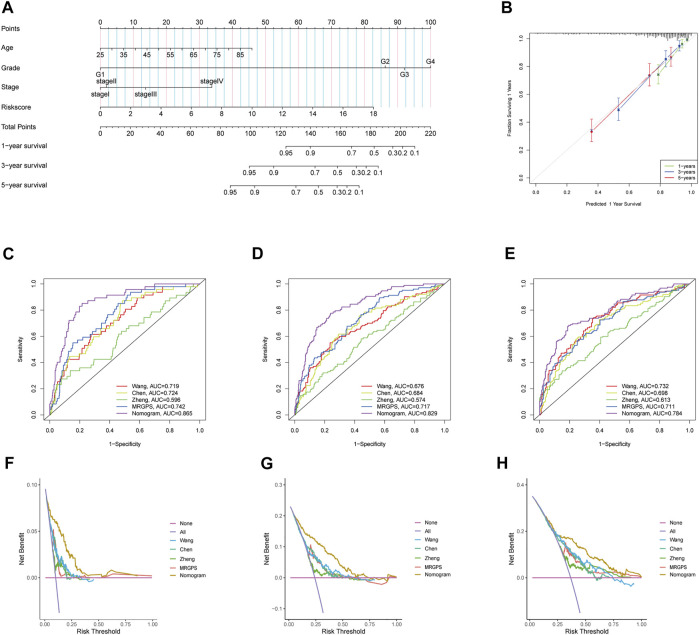
Construction and verification of nomogram. **(A)** The prognostic nomogram constructed based on the risk score of MRGPS and clinicopathological parameters predicted the survival rate of TCGA-ccRCC patients at 1-, 3-, and 5-years. **(B)** Calibration curves showed the concordance between predicted and observed 1-, 3-, and 5-years survival rates. AUCs of the nomogram, MRGPS and other signatures in ROC analysis were calculated at **(C)** 1-, **(D)** 3-, and **(E)** 5-years OS time in TCGA-ccRCC cohort. Decision curve analyses (DCA) for nomogram, MRGPS and other signatures at **(F)** 1-, **(G)** 3-, and **(H)** 5-years to assess clinical utility in TCGA-ccRCC cohort.

Furthermore, we discovered that the current risk score was an independent risk factor for OS in the training, validation, and whole cohorts (*p* < 0.05, Table 2). Subsequently, we developed a nomogram based on age, differentiation grade, stage, and MRGPS risk score to further predict the OS of ccRCC patients belonging to the TCGA cohort (*p* < 0.1, Figure 8A). We observed good calibrations regarding the predicted vs observed 1-, 3-, and 5-years OS of the patients (Figure 8B). Moreover, the ROC curves exhibited better predictive capability in the current nomogram to predict the 1-, 3-, and 5-years OS than the MRGPS and risk scores published by Wang et al. (2021), Chen et al. (2021), and Zheng et al. (2021) (Figures 8C–E). Additionally, DCA analysis revealed the superiority of the current nomogram over MRGPS and the published risk scores in predicting the 1-, 3-, and 5-years OS (Figures 8F–H).

**TABLE 2:** Multivariate Cox regression analysis in training, validation, and the whole cohorts.

Characteristics	Multivariate analysis		
Hazard ratio (95% CI)	Training cohort	Validation cohort	The whole cohorts
Age	1.045 (1.019–1.072)^a^	1.027 (1.009–1.046)^a^	1.032 (1.017–1.047)^a^
Gender	0.955 (0.554–1.649)	0.879 (0.577–1.340)	0.958 (0.691–1.329)
Grade	1.168 (0.761–1.791)	1.474 (1.098–1.978)^a^	1.398 (1.108–1.765)^a^
Stage	2.123 (0.955–4.722)	1.504 (0.834–2.710)	1.559 (0.985–2.466)
T	0.687 (0.339–1.390)	0.914 (0.530–1.577)	0.894 (0.589–1.357)
M	1.561 (0.499–4.886)	1.426 (0.593–3.428)	1.523 (0.774–2.996)
Risk score	1.218 (1.115–1.332)^a^	1.230 (1.076–1.406)^a^	1.222 (1.141–1.308)^a^

a Statistically significant (*p* < 0.05).

### Validation Using Quantitative Real-Time Transcription-PCR and Human Protein Atlas Datasets

**FIGURE 9:**
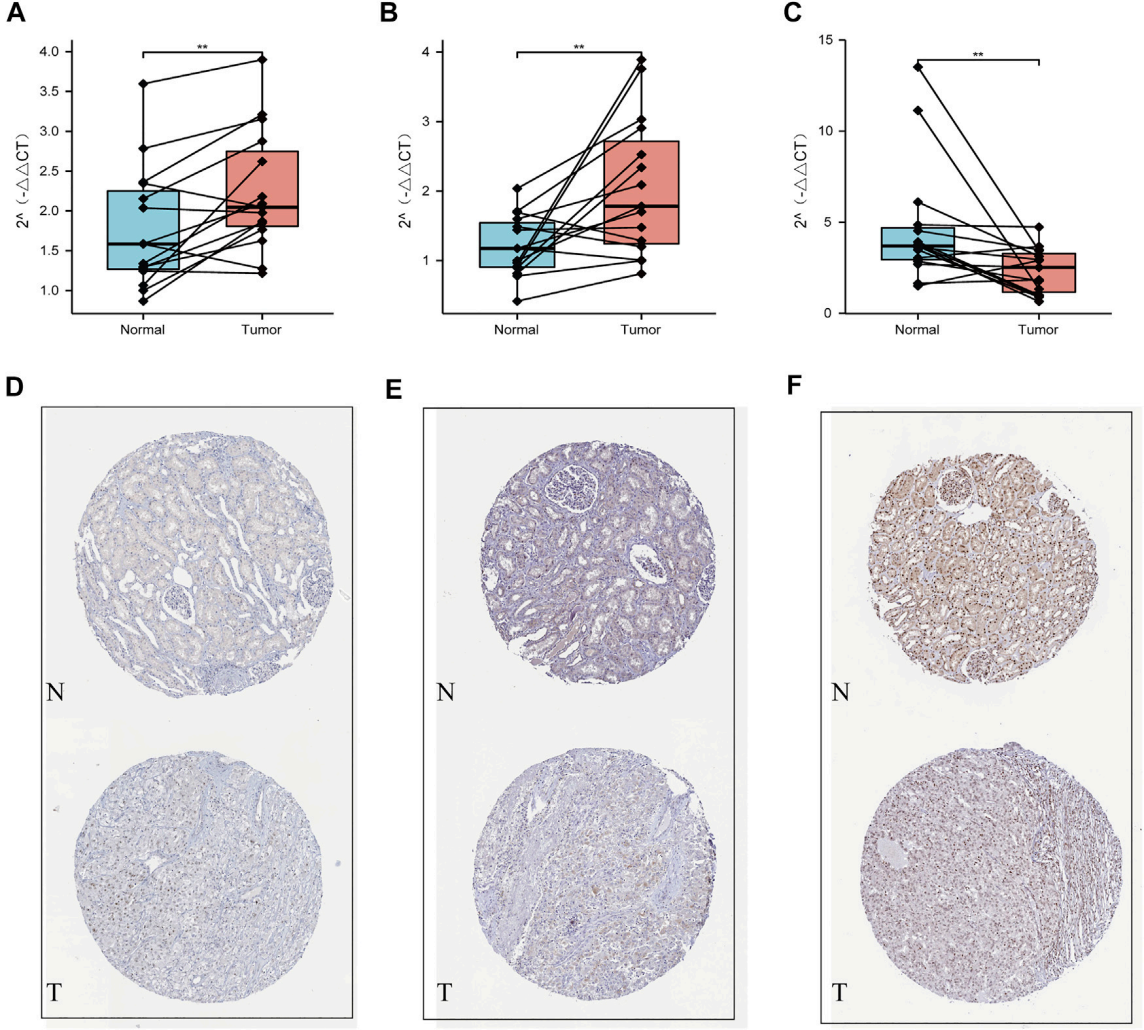
Validation using qPCR and HPA datasets. **(A)** NOP2, **(B)** NSUN6, and **(C)** TET2 mRNA expression measured by qRT-PCR. Validation of the differences in expression of **(D)** NOP2, **(E)** NSUN6, and **(F)** TET2 between renal cancer and normal renal tissue at the translational level with data from the HPA database.

Our qRT-PCR analysis revealed elevated expression levels of NOP2 and NSUN6, but decreased expression of TET2 were in the tumor tissues compared to those in the paired normal tissues of 15 ccRCC samples obtained from FPH (*p* < 0.05, Figures 9A–C). The results of HPA database demonstrated that the expression levels of both NOP2 and NSUN6 were higher in the ccRCC tissues than those in the normal tissues; however, the expression of TET2 was significantly lower in the ccRCC tissues than that in the normal tissue (Figures 9D–F).

## Discussion

Global and local changes in DNA/RNA/histone methylation are seminal features of malignant tumor cells (Michalak et al., 2019). In the current study, we identified three MRGs (*NOP2*, *NSUN6*, and *TET2*) from TCGA data and established an MRGPS for the prognoses of ccRCC patients. This MRGPS exhibited excellent calibration and discrimination. In addition, we validated the three candidate genes in 15 paired ccRCC samples obtained from FPH by qRT-PCR. Furthermore, the current risk score was correlated with tumor immune microenvironment characteristics and could be used as a potential biomarker of ccRCC response to ICIs.

Of note, ccRCC is a highly heterogeneous malignancy (Jonasch et al., 2021). The existing prognosis models that incorporate clinicopathological characteristics, such as the AJCC staging system and the Mayo Clinic stage and necrosis score, have improved prognosis capacity (Parker et al., 2017). However, owing to the complex molecular mechanism of ccRCC, clinical parameters alone are inadequate for predicting the prognoses of ccRCC patients. Interestingly, chromatin methylations, such as m5C and m6A, play a fundamental role in the ccRCC carcinogenesis (Angulo et al., 2021). Nonetheless, comprehensive exploration of chromatin methylation in ccRCC is still lacking. In this study, we established a novel MRGPS using data from TCGA ccRCC patients; this MRGPs improved the prognoses of ccRCC patients with a C-index as high as 0.798 at 1-year OS. In addition, close links were identified between the clinical and pathological characteristics of ccRCC and MRGPS: age, sex, differentiation, and tumor node metastasis (TNM) stage. Furthermore, a MRGPS-incorporating nomogram demonstrated a higher prognostic capacity and clinical utility than published risk scores.

Among the three MRGs identified, *NOP2* and *NSUN6* were prognostic risk factors, whereas *TET2* was a prognostic protective factor. Notably, *NOP2* and *NSUN6* are key members of the NOP2/Sun domain family and possess S-adenosyl-L-methionine-dependent methyltransferase activity (Frye and Blanco, 2016). *NOP2* is upregulated in various cancers, including lung adenocarcinoma, breast cancer, and prostate cancer, and it is associated with tumor aggressiveness (Ma et al., 2017). Deficiency of *NSUN6*-mediated methylation can downregulate transcription and translation. While *NSUN6* expression is highest in the testis and lowest in the blood, it is heterogeneous in different tumors. However, it is downregulated in tumors originating from tissues that have high *NSUN6* expression, such as the testis, thyroid, and ovaries (Selmi et al., 2021). In contrast, it is upregulated in tumors originating from tissues that have low *NSUN6* expression, such as that in hematologic tumor and kidney cancer. Moreover, *NSUN6* is associated with prognosis of various cancers, including pancreatic cancer (Yang et al., 2021) and hepatocellular carcinoma (Wang et al., 2018). It also plays an important role in bone metastasis (Li et al., 2017). On the other hand, *TET2* mutations have been widely identified various myeloid malignancies. In fact, *TET2* inactivation leads to polyhematopoietic abnormalities in mice, which is a recurrent event in human lymphoma formation (Ferrone et al., 2020). Notably, *TET2* dysfunction mutations are generally associated with DNA hypermethylation, tumor progression, and poor patient outcomes (Cimmino et al., 2017). However, *NOP2, NSUN6,* and *TET2* have been rarely studied in ccRCC. In the present study, qRT-PCR data from 15 paired FPH ccRCC samples revealed that while *NOP2* and *NSUN6* were upregulated, *TET2* was downregulated in tumor tissues compared with those in normal tissues. In summary, *NOP2*, *NSUN6*, and *TET2* were identified as prognostic biomarkers for ccRCC; however, additional *in vitro* and *in vivo* research is needed to validate these findings.

The potential mechanisms of MRGPS regulating ccRCC prognosis deserved further study. In the present study, we found via KEGG analysis and GSEA that the PI3K-AKT and mTOR signaling pathways were highly enriched in the high-risk subgroup. The PI3K signaling pathway facilitates several essential cellular functions, such as cell proliferation, growth, migration, metabolism, and survival (Fruman and Rommel, 2014). In a large cohort of 419 primary ccRCC patients, aberrantly expressed components of the PI3K signaling cascade (e.g., PTEN, PI3K, p-AKT, mTOR, p-mTOR, p-S6, and p-4EBP1 proteins) exhibited aggressive pathological features and caused adverse survival (Darwish et al., 2013). Therefore, we hypothesized that poor prognoses of patients with a high MRGPS might be because of activation of the PI3K-AKT and mTOR pathways; nonetheless, this hypothesis requires further exploration.

Since ccRCC is a highly vascular tumor, the levels of angiogenic factors, including VEGF, are correlated with its prognosis (Choueiri and Kaelin, 2020). Inhibition of VEGFR generally causes vascular normalization, thereby activating anti-tumor immunity (Hsieh et al., 2017). Until 2017, the multikinase inhibitors sunitinib and pazopanib that primarily target VEGFR formed the frontline treatment for ccRCC (Powles et al., 2021). The median progress free survival (PFS), OS and ORR for sunitinib and pazopanib are 8.4 and 9.5 months, 28.4 months and 29.3 months, and 25 and 31%, respectively (George et al., 2021). The complexity of the VEGFR family may possibly be responsible for the inconsistent results. In this study, we found that the current MRGPS could be used as an alternative to VEGFRs. Remarkably, it had a positively correlation with VEGFB/D and negative correlation with VEGFC; however, it did not have a correlation with VEGFA. Furthermore, we also found that while sunitinib had a lower IC_50_, pazopanib had a higher IC_50_ in high-risk patients than those in low-risk patients, according to the current MRGPS. This highlighted the response divergence between sunitinib and pazopanib and clarified personalized TKI treatment for ccRCC patients.

Although ccRCC patients have a typically suppressed immune status, they are highly abundant in immune cells (Şenbabaoğlu et al., 2016). In this study, we revealed that the current MRGPS was correlated with tumor-infiltrating lymphocytes: high MRGPS was associated with increased number of CD8+T cell number, activated NK cell, follicular helper cells T cells, and regulatory T cells. In contrast, low MRGPS was associated with decreased number of naïve B cells, resting memory CD4^+^ T cells, monocytes, macrophages M2, and resting mast cells, as previously reported (Díaz-Montero et al., 2020). In addition, the current MRGPS was also associated with co-stimulatory molecules, such as CD40 and CD58. Immune checkpoints are cell surface receptors expressed on immune cells, and their inhibition causes immune activation. In the present study, we found that high MRGPS was associated with PDCD1 and CTLA4 expression levels (both *p* < 0.001). Further analysis revealed that low MRGPS was correlated with lower TIDE score and higher MSI score than high MRGPS (both *p* < 0.05), indicating that patients with low MRGPS would benefit more from ICIs. Importantly, this finding was validated in 298 IMvigor patients receiving atezolizumab. Therefore, MRGPS is a promising biomarker for predicting the response of ccRCC patients towards ICIs.

Nonetheless, our study has several limitations. First, both the data from TCGA and FPH are retrospective; therefore, the risk score needs to be verified in prospective cohorts. Second, merely incorporating MRGPS to build a prognostic model is inadequate, regardless of its importance. Third, samples from FPH were too few, and the results need to be validated in more samples*.* Finally, the associations of the current MRGPS with tumor mutations, tumor immune microenvironment, and TKI and ICI responses require further validation *in vitro* and *in vivo*.

In conclusion, the current MRGPS consisting of *NOP2, NSUN6,* and *TET2* is a potential alternative prognostic biomarker for ccRCC patients and is also be a promising index for personalized ICI treatments in ccRCC.

## Data Availability

Publicly available datasets were analyzed in this study. This data can be found here: Expression profile data are available in The Cancer Genome Atlas (TCGA) (https://portal.gdc.cancer.gov/). Immunohistochemical staining of genes are available in The Human Protein Atlas (HPA) (http://www.proteinatlas.org/). And the experimental data of qRT-RCR can be downloaded from the supplementary file.

## Supplementary Material

Supplementary Figure S1 The ccRCC patients from TCGA was divided into different clusters when k=3-9 **(A–G)**. Relative change in area under CDF curve for k=2 to 9 **(H)**.

Supplementary Figure S2 The correlation analysis with clinical characteristic. **(A)** Bargraph demonstrates the proportion of patients in the high- and low- risk groups with different clinicopathological characteristics. **(B-M)** Kaplan-Meier analysis of subgroup patients based on risk scores.
