# Supplementary material for: Identification of a Methylation-Regulating Genes Prognostic Signature to Predict the Prognosis and Aid Immunotherapy of Clear Cell Renal Cell Carcinoma
Source: Front Cell Dev Biol. 2022 Mar 2;10:832803. doi: 10.3389/fcell.2022.832803 (PMC8924039; doi:10.3389/fcell.2022.832803)
Supplement: Supplementary file 1 [file Table4.DOC]

S Table 4. Clinical characteristics of the ccRCC patients in training cohort and Validation cohort.

| Characteristic | Training cohort | Validation cohort | p |
| --- | --- | --- | --- |
| n | 160 | 366 |  |
| age, meidan (IQR) | 60 (52, 69.25) | 61 (51, 69) | 0.966 |
| gender, n (%) |  |  | 0.573 |
| FEMALE | 59 (11.2%) | 124 (23.6%) |  |
| MALE | 101 (19.2%) | 242 (46%) |  |
| grade, n (%) |  |  | 0.885 |
| G1 | 3 (0.6%) | 10 (1.9%) |  |
| G2 | 65 (12.4%) | 161 (30.6%) |  |
| G3 | 64 (12.2%) | 141 (26.8%) |  |
| G4 | 26 (4.9%) | 48 (9.1%) |  |
| GX | 1 (0.2%) | 4 (0.8%) |  |
| unknow | 1 (0.2%) | 2 (0.4%) |  |
| stage, n (%) |  |  | 0.689 |
| Stage I | 75 (14.3%) | 186 (35.4%) |  |
| Stage II | 18 (3.4%) | 39 (7.4%) |  |
| Stage III | 41 (7.8%) | 82 (15.6%) |  |
| Stage IV | 26 (4.9%) | 56 (10.6%) |  |
| unknow | 0 (0%) | 3 (0.6%) |  |
| T, n (%) |  |  | 0.647 |
| T1 | 76 (14.4%) | 191 (36.3%) |  |
| T2 | 20 (3.8%) | 49 (9.3%) |  |
| T3 | 60 (11.4%) | 119 (22.6%) |  |
| T4 | 4 (0.8%) | 7 (1.3%) |  |
| M, n (%) |  |  | 0.659 |
| M0 | 130 (24.7%) | 288 (54.8%) |  |
| M1 | 23 (4.4%) | 55 (10.5%) |  |
| MX | 7 (1.3%) | 23 (4.4%) |  |
| N, n (%) |  |  | 0.805 |
| N0 | 72 (13.7%) | 166 (31.6%) |  |
| N1 | 6 (1.1%) | 10 (1.9%) |  |
| NX | 82 (15.6%) | 190 (36.1%) |  |
