# Supplementary figures and images for: Identification of a Methylation-Regulating Genes Prognostic Signature to Predict the Prognosis and Aid Immunotherapy of Clear Cell Renal Cell Carcinoma
Source: Front Cell Dev Biol. 2022 Mar 2;10:832803. doi: 10.3389/fcell.2022.832803 (PMC8924039; doi:10.3389/fcell.2022.832803)

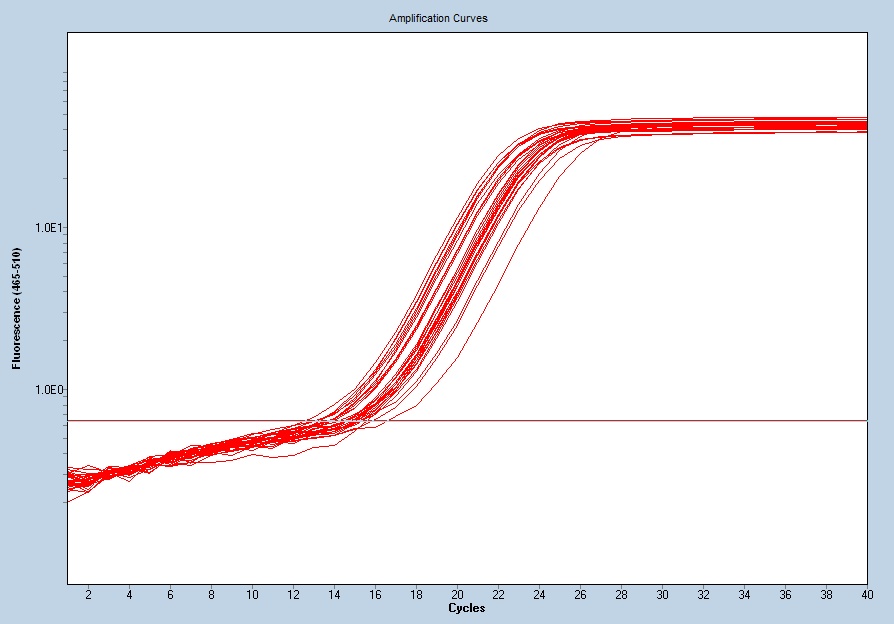

Supplement: Supplementary file 2 [file DataSheet1.ZIP › 实验数据/扩增及溶解曲线/actin-1-a.jpg]

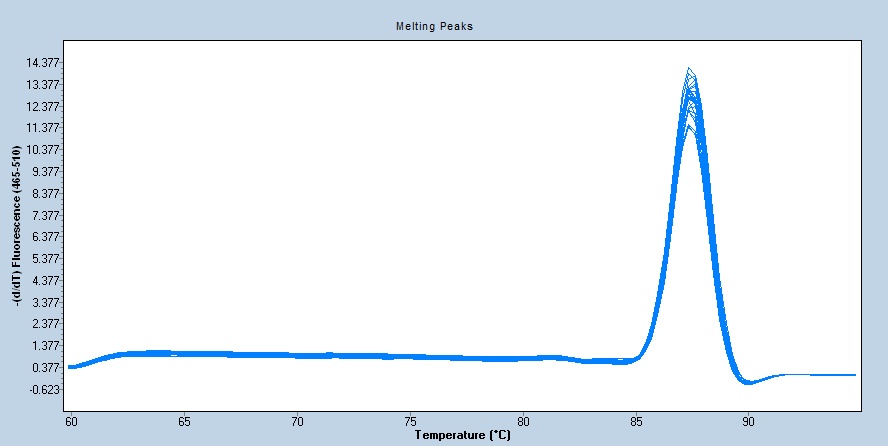

Supplement: Supplementary file 2 [file DataSheet1.ZIP › 实验数据/扩增及溶解曲线/actin-1-m.jpg]

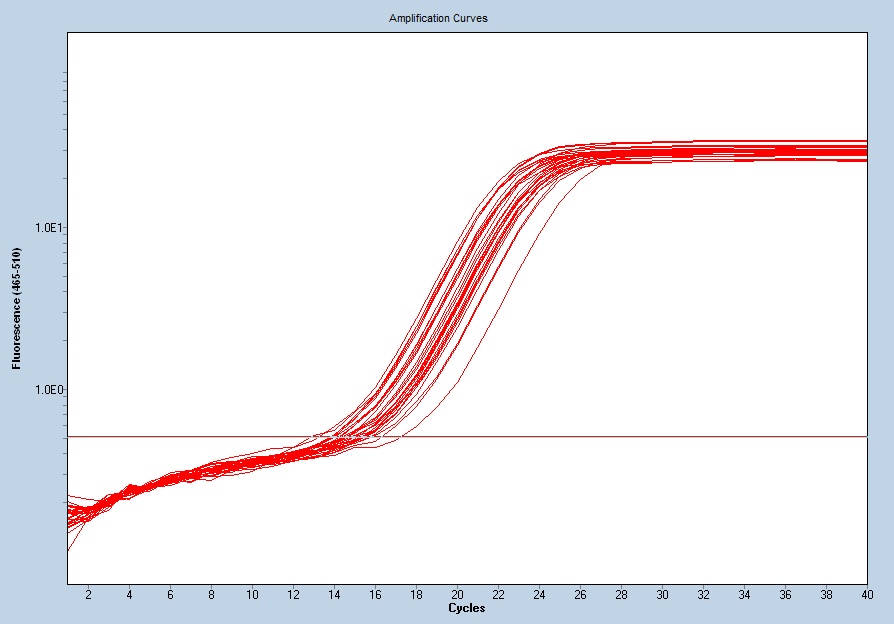

Supplement: Supplementary file 2 [file DataSheet1.ZIP › 实验数据/扩增及溶解曲线/actin-2-a.jpg]

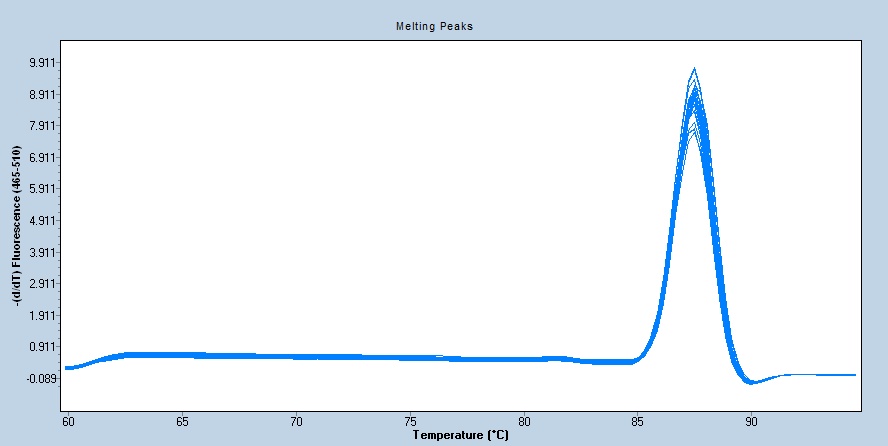

Supplement: Supplementary file 2 [file DataSheet1.ZIP › 实验数据/扩增及溶解曲线/actin-2-m.jpg]

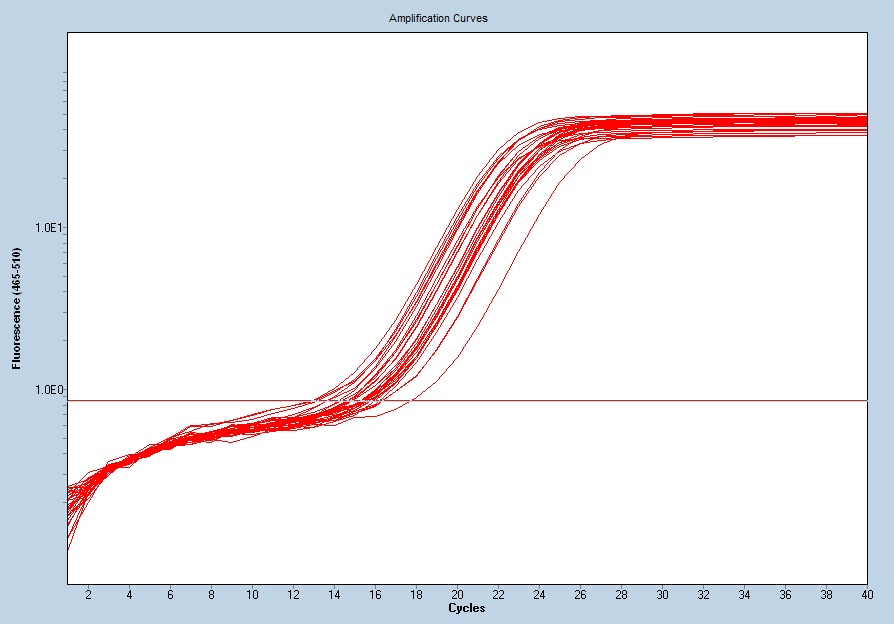

Supplement: Supplementary file 2 [file DataSheet1.ZIP › 实验数据/扩增及溶解曲线/actin-3-a.jpg]

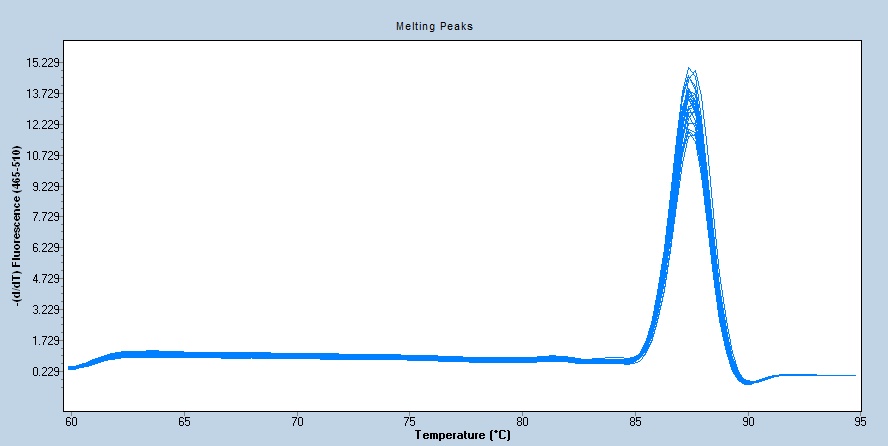

Supplement: Supplementary file 2 [file DataSheet1.ZIP › 实验数据/扩增及溶解曲线/actin-3-m.jpg]

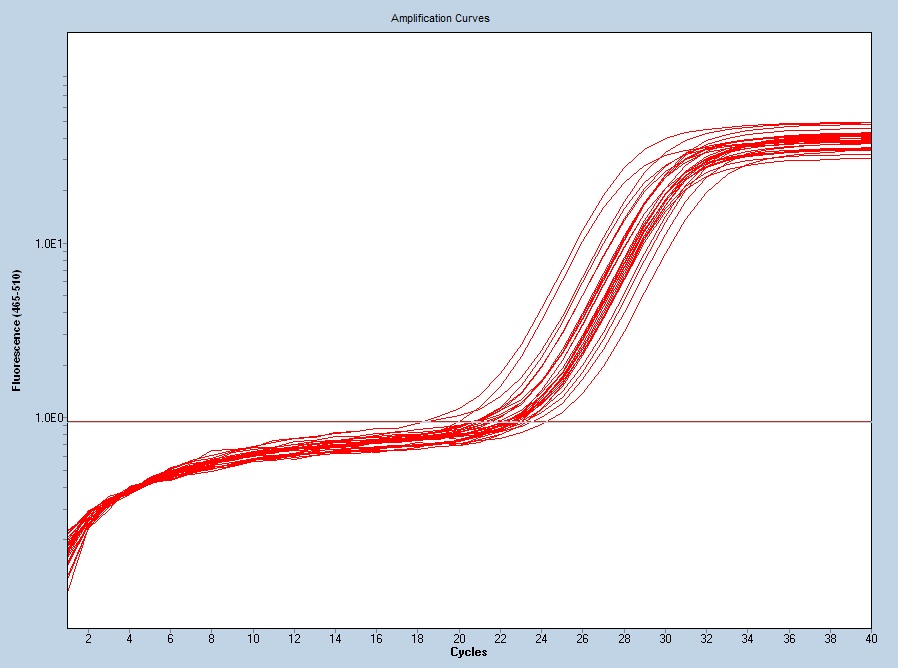

Supplement: Supplementary file 2 [file DataSheet1.ZIP › 实验数据/扩增及溶解曲线/NOP2-1-A.jpg]

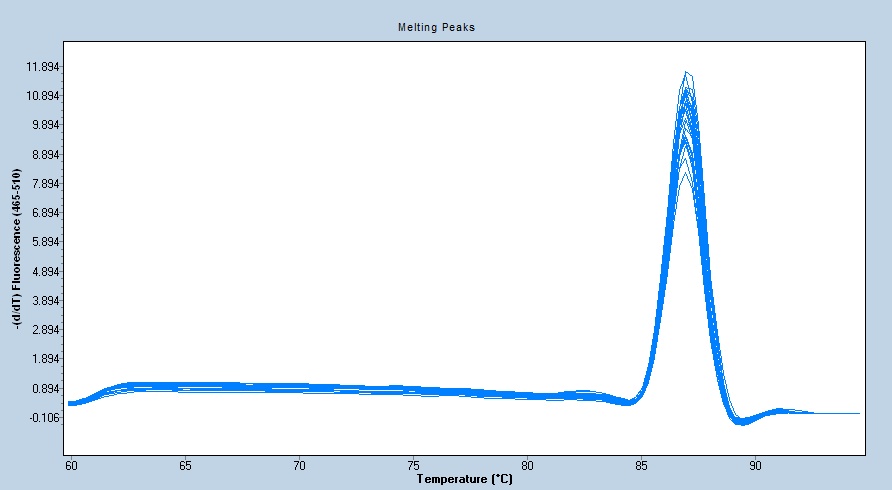

Supplement: Supplementary file 2 [file DataSheet1.ZIP › 实验数据/扩增及溶解曲线/NOP2-1-M.jpg]

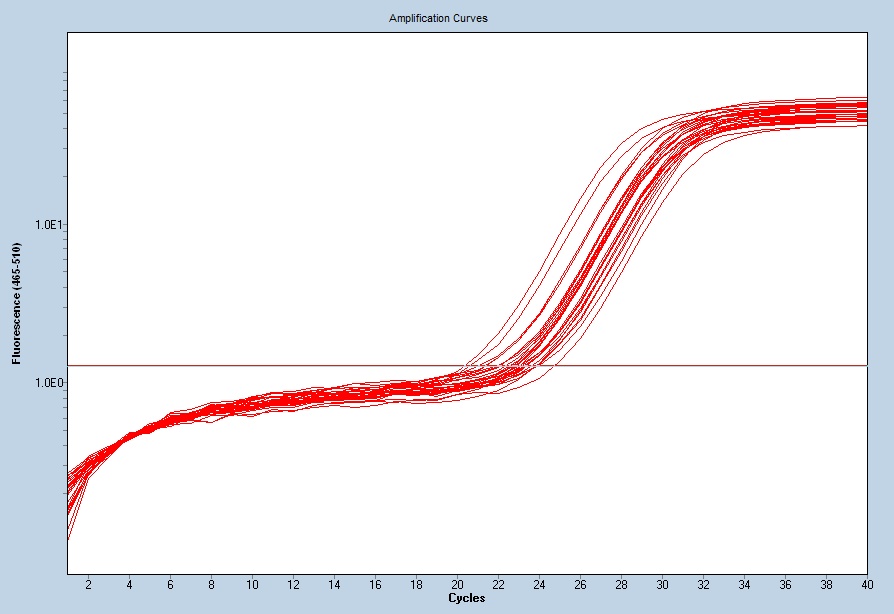

Supplement: Supplementary file 2 [file DataSheet1.ZIP › 实验数据/扩增及溶解曲线/NOP2-2-A.jpg]

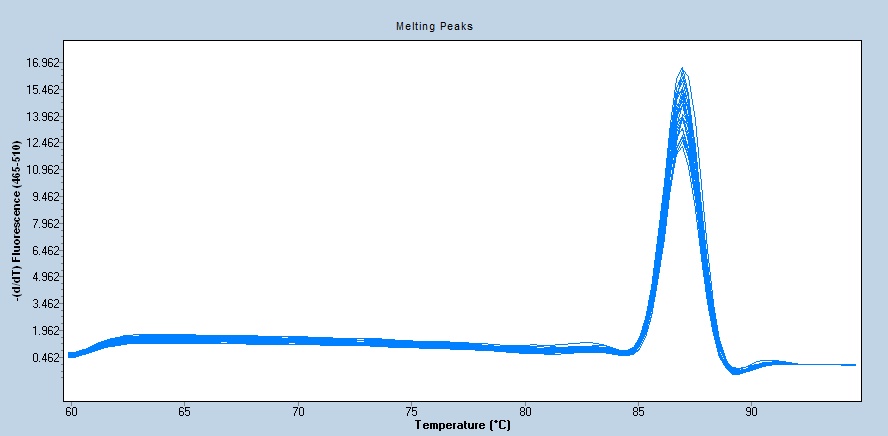

Supplement: Supplementary file 2 [file DataSheet1.ZIP › 实验数据/扩增及溶解曲线/NOP2-2-M.jpg]

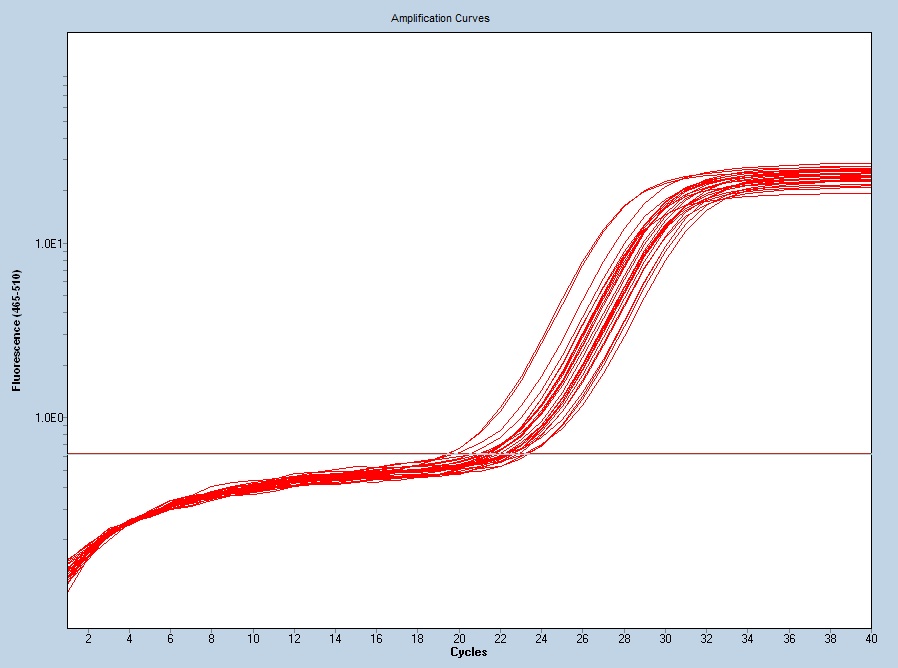

Supplement: Supplementary file 2 [file DataSheet1.ZIP › 实验数据/扩增及溶解曲线/NOP2-3-A.jpg]

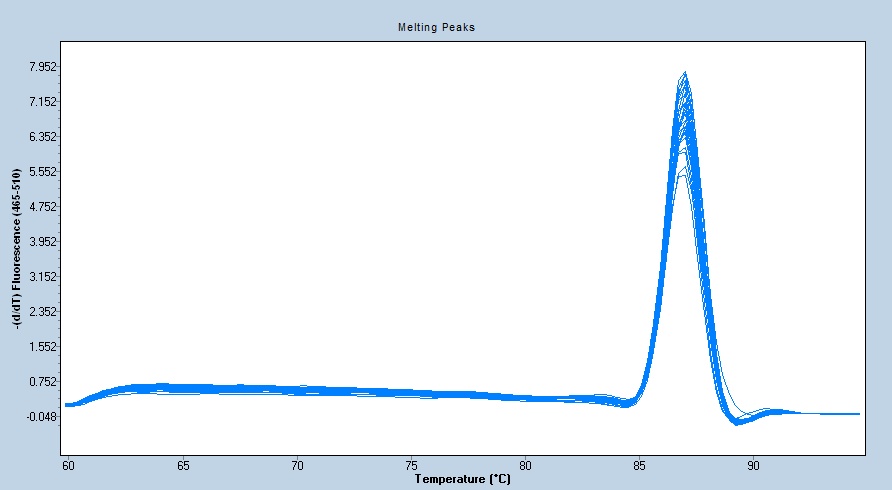

Supplement: Supplementary file 2 [file DataSheet1.ZIP › 实验数据/扩增及溶解曲线/NOP2-3-M.jpg]

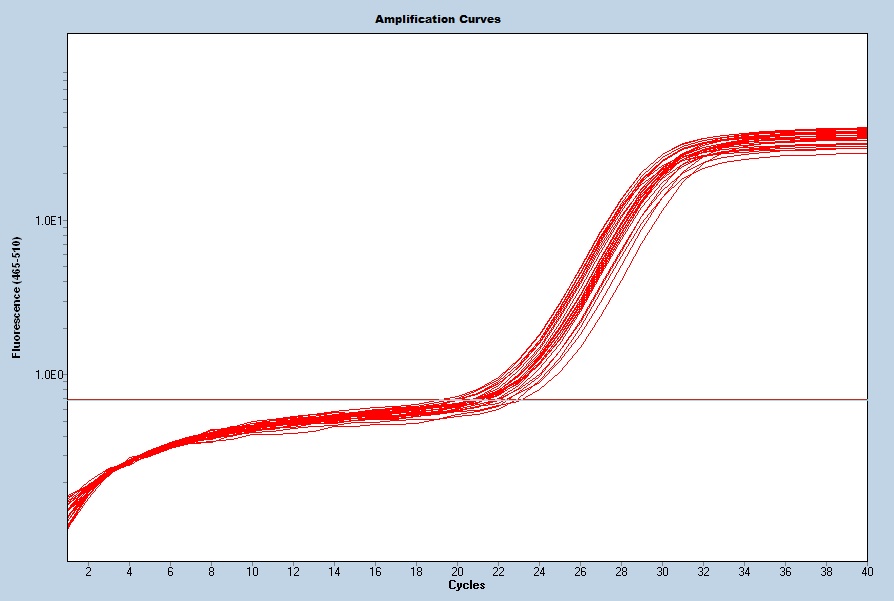

Supplement: Supplementary file 2 [file DataSheet1.ZIP › 实验数据/扩增及溶解曲线/NSUN6-1-A.jpg]

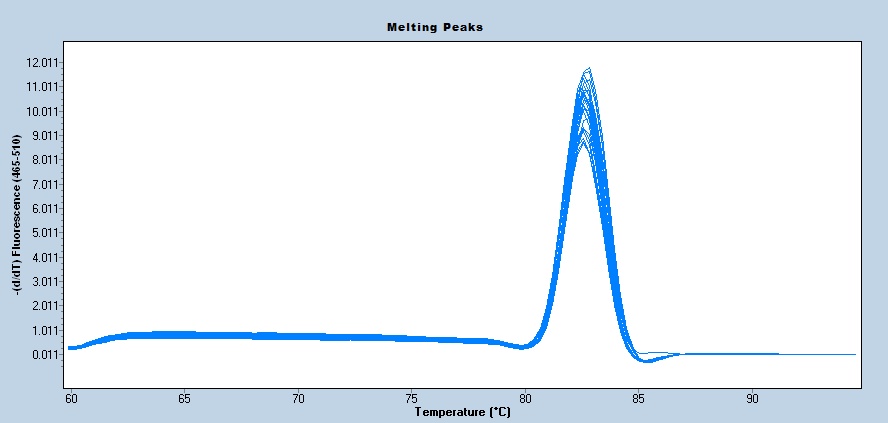

Supplement: Supplementary file 2 [file DataSheet1.ZIP › 实验数据/扩增及溶解曲线/NSUN6-1-M.jpg]

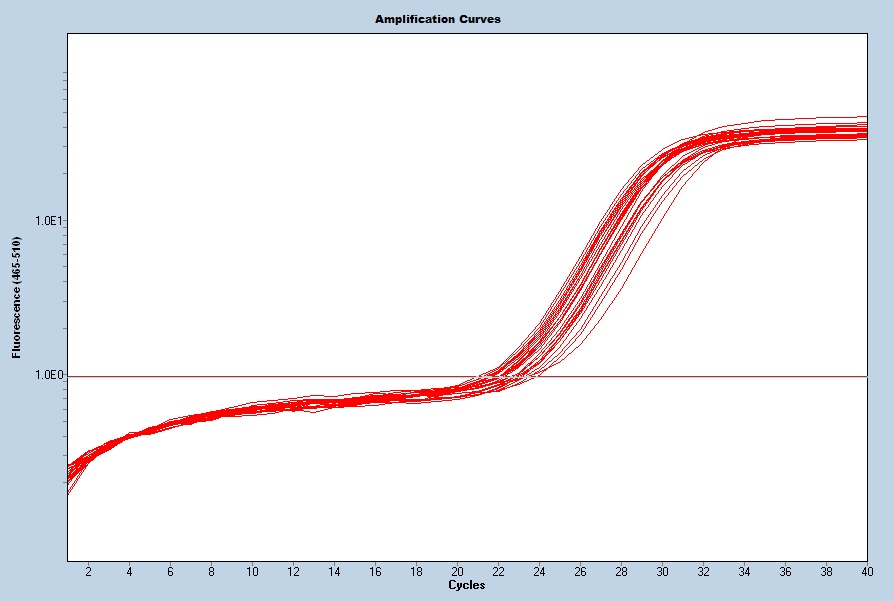

Supplement: Supplementary file 2 [file DataSheet1.ZIP › 实验数据/扩增及溶解曲线/NSUN6-2-A.jpg]

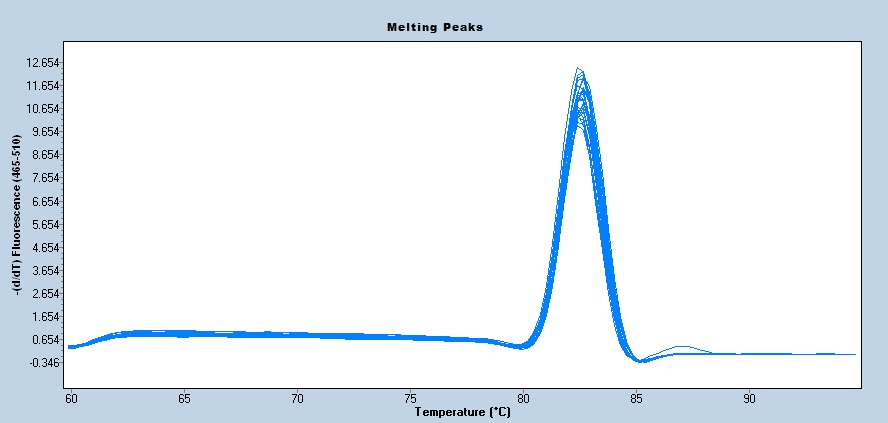

Supplement: Supplementary file 2 [file DataSheet1.ZIP › 实验数据/扩增及溶解曲线/NSUN6-2-M.jpg]

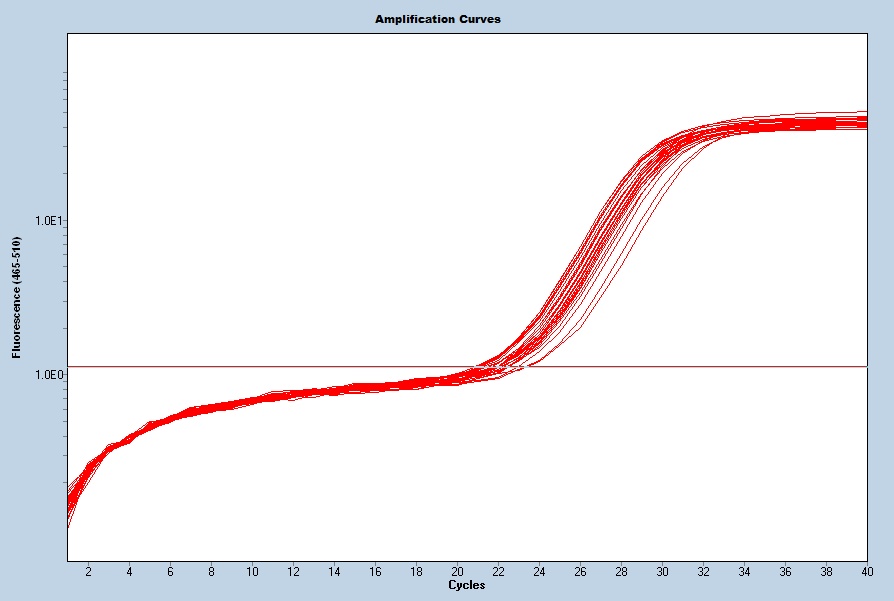

Supplement: Supplementary file 2 [file DataSheet1.ZIP › 实验数据/扩增及溶解曲线/NSUN6-3-A.jpg]

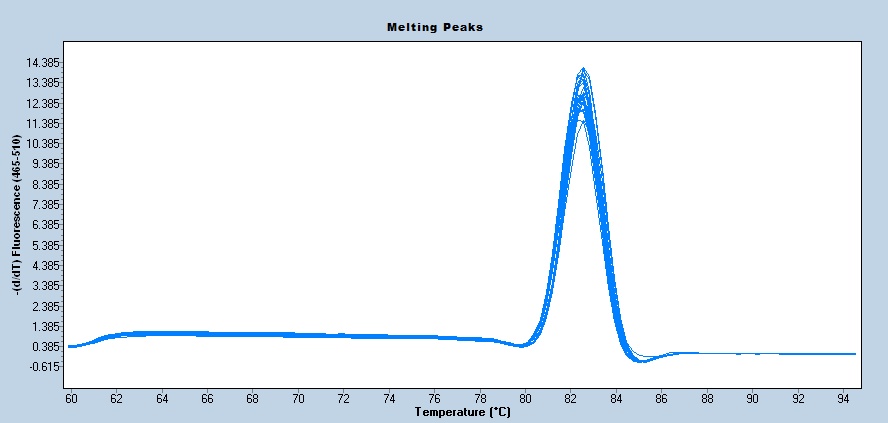

Supplement: Supplementary file 2 [file DataSheet1.ZIP › 实验数据/扩增及溶解曲线/NSUN6-3-M.jpg]

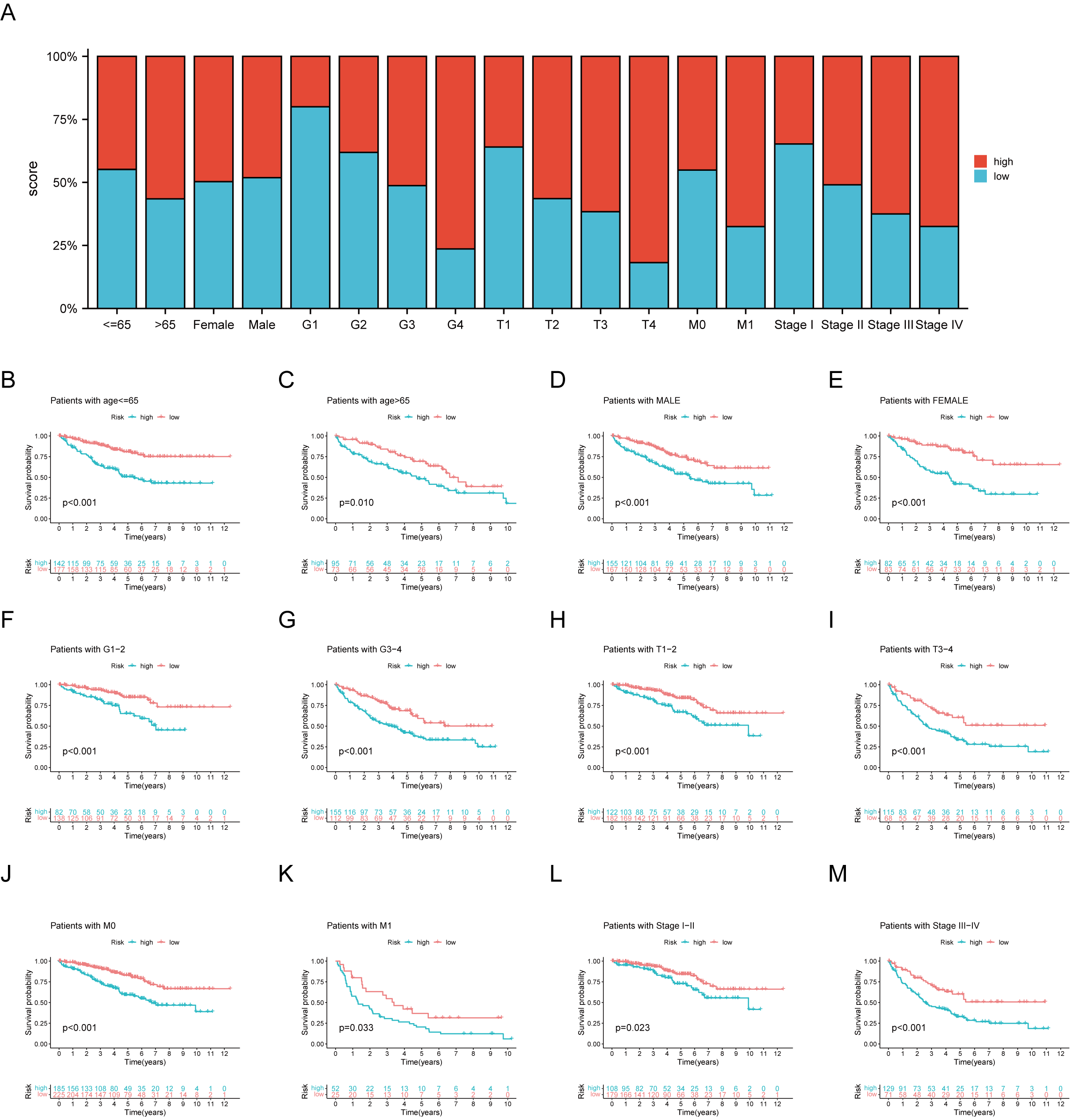

Supplement: Supplementary file 3 [file Image2.TIF]

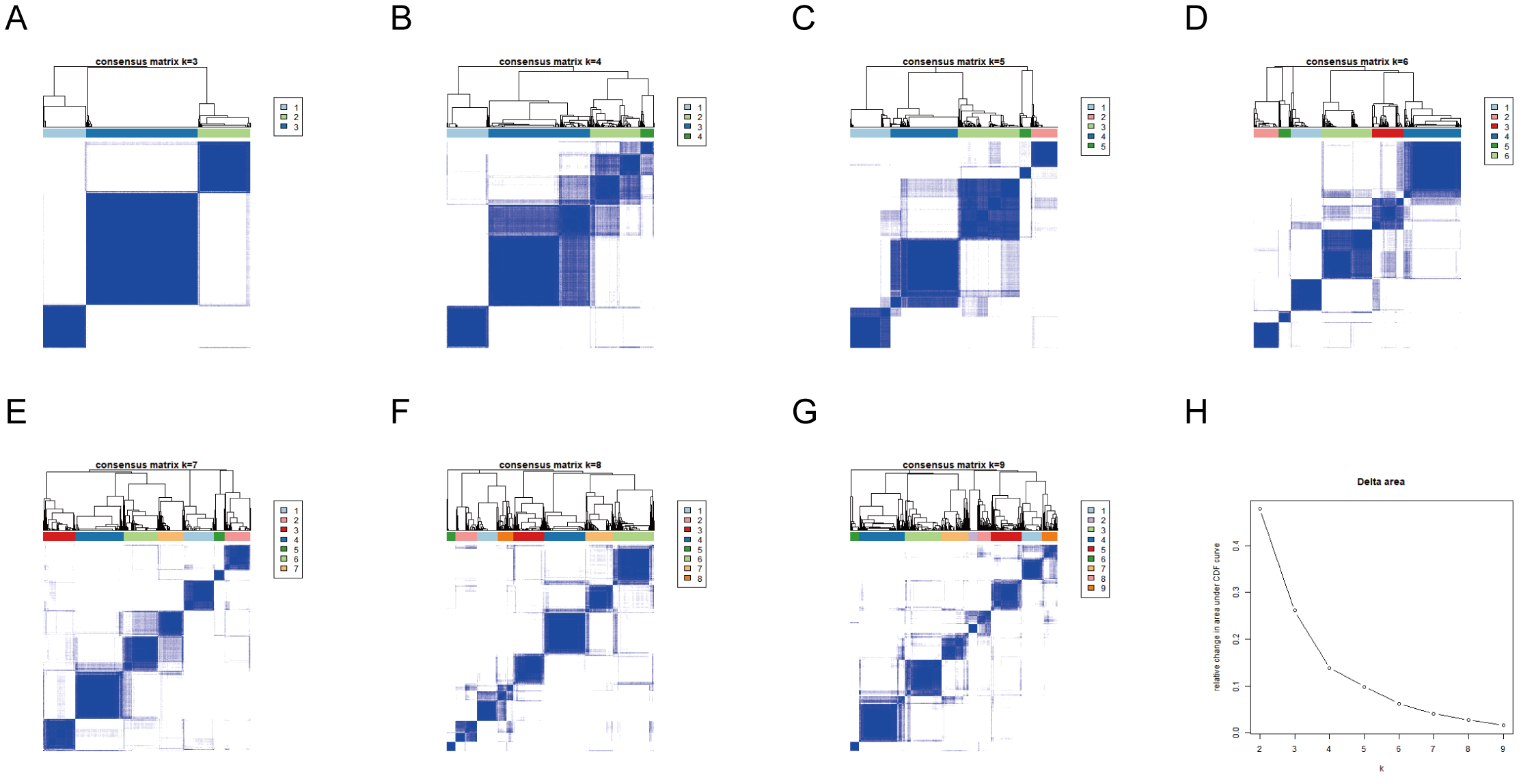

Supplement: Supplementary file 4 [file Image1.TIF]
