# Supplementary material for: Identification of a Methylation-Regulating Genes Prognostic Signature to Predict the Prognosis and Aid Immunotherapy of Clear Cell Renal Cell Carcinoma
Source: Front Cell Dev Biol. 2022 Mar 2;10:832803. doi: 10.3389/fcell.2022.832803 (PMC8924039; doi:10.3389/fcell.2022.832803)
Supplement: Supplementary file 5 [file Table1.DOC]

**S table1.** The methylation-regulated genes set from GSEA.

| AASS | CBLL1 | DYDC1 | FOS | KDM6A | METTL16 |
| --- | --- | --- | --- | --- | --- |
| AHCY | CHD5 | DYDC2 | FTSJ1 | KMT2A | METTL17 |
| ALKBH8 | CHTOP | ECE2 | FTSJ3 | KMT2B | METTL18 |
| ANTKMT | CMTR1 | EED | GAMT | KMT2C | METTL21A |
| ARID4A | CMTR2 | EEF1AKMT1 | GATA3 | KMT2D | METTL21C |
| ARMT1 | COMT | EEF1AKMT2 | GATAD2A | KMT2E | METTL21EP |
| AS3MT | COMTD1 | EEF1AKMT3 | GCG | KMT5A | METTL22 |
| ASH1L | COPRS | EEF1AKMT4 | GFI1 | KMT5B | METTL23 |
| ASH2L | COQ3 | EEF1AKMT4-ECE2 | GNAS | KMT5C | METTL24 |
| ASMT | COQ5 | EEF1AKNMT | GNMT | LARP7 | METTL25 |
| ASMTL | CREBBP | EEF2KMT | GRHL2 | LCMT1 | METTL2A |
| ASZ1 | CSKMT | EHMT1 | GSK3A | LCMT2 | METTL2B |
| ATF7IP | CTCF | EHMT2 | GSPT1 | LRTOMT | METTL3 |
| ATPSCKMT | CTCFL | EMG1 | GSTO1 | MACROH2A1 | METTL4 |
| ATRX | CTNNB1 | ETF1 | GTPBP3 | MAEL | METTL5 |
| AUTS2 | CTR9 | ETFBKMT | H1-5 | MAPT-AS1 | METTL6 |
| BAZ2A | CXXC1 | EZH1 | H1-8 | MAT1A | METTL7A |
| BCDIN3D | CYP1A1 | EZH2 | HELLS | MAT2A | METTL7B |
| BCOR | CYP1A2 | EZHIP | HEMK1 | MAT2B | METTL8 |
| BEND3 | DDX4 | FAM86B1 | HENMT1 | MBD2 | MGMT |
| BHMT | DIMT1 | FAM86B2 | HNMT | MBD3 | MIR29A |
| BHMT2 | DMAP1 | FAM86C1P | HSD17B10 | MECOM | MIR29B1 |
| BMT2 | DNMT1 | FAM86C2P | ICMT | MECP2 | MIR29C |
| BRCA1 | DNMT3A | FAM98A | INMT | MEN1 | MIS18A |
| BRD4 | DNMT3B | FAM98B | IWS1 | MEPCE | MLLT6 |
| BTG2 | DNMT3L | FBL | JARID2 | METTL1 | MORC1 |
| BUD23 | DOT1L | FBLL1 | KDM1A | METTL11B | MOV10L1 |
| CAMKMT | DPH5 | FBXO11 | KDM1B | METTL14 | MPHOSPH8 |
| CARM1 | DPPA3 | FDXACB1 | KDM3A | METTL15 | MRM1 |
| CARNMT1 | DPY30 | FKBP6 | KDM4D | METTL15P1 | MRM2 |
| MRM3 | OGT | PRDM2 | RTF1 | SUV39H1 | TRMT13 |
| MTA2 | PAF1 | PRDM4 | SETD1A | SUV39H2 | TRMT1L |
| MTERF4 | PAGR1 | PRDM5 | SETD1B | SUZ12 | TRMT2A |
| MTF2 | PARP1 | PRDM6 | SETD2 | TARBP1 | TRMT2B |
| MTHFR | PAX5 | PRDM7 | SETD3 | TDRD1 | TRMT44 |
| MTO1 | PAXBP1 | PRDM8 | SETD4 | TDRD12 | TRMT5 |
| MTR | PAXIP1 | PRDM9 | SETD5 | TDRD5 | TRMT6 |
| MTRR | PCIF1 | PRMT1 | SETD6 | TDRD9 | TRMT61A |
| MYB | PCMT1 | PRMT2 | SETD7 | TDRKH | TRMT61B |
| MYC | PCMTD1 | PRMT3 | SETD9 | TET1 | TRMT9B |
| N6AMT1 | PCMTD2 | PRMT5 | SETDB1 | TET2 | TTLL12 |
| NDUFAF5 | PEMT | PRMT6 | SETDB2 | TET3 | TYMS |
| NDUFAF7 | PHF1 | PRMT7 | SETMAR | TEX15 | TYW3 |
| NELFE | PHF19 | PRMT8 | SIRT1 | TFB1M | VCPKMT |
| NIBAN2 | PICK1 | PRMT9 | SIRT7 | TFB2M | VIRMA |
| NNMT | PIH1D1 | PRORP | SMAD4 | TGS1 | WDR4 |
| NOP2 | PIK3CA | PWP1 | SMARCB1 | THADA | WDR5 |
| NR1H4 | PIWIL2 | PYGO2 | SMYD1 | THUMPD2 | WDR5B |
| NSD1 | PIWIL4 | RAB3D | SMYD2 | THUMPD3 | WDR61 |
| NSD2 | PLD6 | RAB6A | SMYD3 | TPMT | WDR82 |
| NSD3 | PNMT | RAMAC | SMYD4 | TRDMT1 | WT1 |
| NSUN2 | PPM1D | RBBP5 | SMYD5 | TRIM28 | WTAP |
| NSUN3 | PRDM1 | RBM15 | SNRPB | TRMO | ZC3H13 |
| NSUN4 | PRDM10 | RBM15B | SNRPD3 | TRMT1 | ZCCHC4 |
| NSUN5 | PRDM11 | RIF1 | SNW1 | TRMT10A | ZFP57 |
| NSUN5P1 | PRDM12 | RLF | SPI1 | TRMT10B | ZMPSTE24 |
| NSUN5P2 | PRDM13 | RNF20 | SPOCD1 | TRMT10C | ZNF274 |
| NSUN6 | PRDM14 | RNMT | SPOUT1 | TRMT11 | ZNF304 |
| NSUN7 | PRDM15 | RRNAD1 | STPG4 | TRMT112 | ZNF335 |
| NTMT1 | PRDM16 | RRP8 | SUPT6H | TRMT12 |  |
