# Supplementary material for: Identification of a Methylation-Regulating Genes Prognostic Signature to Predict the Prognosis and Aid Immunotherapy of Clear Cell Renal Cell Carcinoma
Source: Front Cell Dev Biol. 2022 Mar 2;10:832803. doi: 10.3389/fcell.2022.832803 (PMC8924039; doi:10.3389/fcell.2022.832803)
Supplement: Supplementary file 6 [file Table3.DOCX]

**S Table3.** The top 100 biological pathways which enrich in cluster 2 by using GSEA snalysis.

| NAME | ES | NES | NOM p-val | FDR q-val | FWER p-val |
| --- | --- | --- | --- | --- | --- |
| GOBP_INTERFERON_GAMMA_PRODUCTION | 0.6454409 | 2.305793 | 0 | 0.025368357 | 0.035 |
| GOBP_POSITIVE_REGULATION_OF_LEUKOCYTE_PROLIFERATION | 0.5869391 | 2.284442 | 0.002024292 | 0.019927317 | 0.046 |
| GOBP_POSITIVE_REGULATION_OF_INTERFERON_GAMMA_PRODUCTION | 0.68077207 | 2.282519 | 0 | 0.014020666 | 0.046 |
| GOBP_POSITIVE_REGULATION_OF_T_CELL_PROLIFERATION | 0.6370896 | 2.2255275 | 0.002109705 | 0.026528452 | 0.097 |
| GOBP_POSITIVE_REGULATION_OF_INTERLEUKIN_4_PRODUCTION | 0.76484936 | 2.2222047 | 0 | 0.023238301 | 0.101 |
| GOBP_NEGATIVE_REGULATION_OF_LEUKOCYTE_MEDIATED_IMMUNITY | 0.6706056 | 2.2160387 | 0.003913894 | 0.021570792 | 0.105 |
| GOBP_TYPE_2_IMMUNE_RESPONSE | 0.62790465 | 2.2030654 | 0 | 0.022282977 | 0.122 |
| GOBP_NEGATIVE_REGULATION_OF_LYMPHOCYTE_MEDIATED_IMMUNITY | 0.67990184 | 2.201858 | 0.003952569 | 0.019792276 | 0.122 |
| GOBP_REGULATION_OF_TYPE_2_IMMUNE_RESPONSE | 0.63975525 | 2.1959846 | 0 | 0.019015511 | 0.124 |
| GOBP_RESPIRATORY_BURST | 0.73284715 | 2.1949575 | 0.004158004 | 0.017315956 | 0.124 |
| GOBP_DETECTION_OF_EXTERNAL_BIOTIC_STIMULUS | 0.7463475 | 2.191577 | 0.001872659 | 0.016621122 | 0.128 |
| GOBP_ZINC_ION_HOMEOSTASIS | 0.6317859 | 2.1851301 | 0 | 0.017057752 | 0.136 |
| GOBP_RESPONSE_TO_INTERFERON_GAMMA | 0.5862189 | 2.16516 | 0.00409836 | 0.021426827 | 0.158 |
| GOBP_LYMPHOCYTE_MEDIATED_IMMUNITY | 0.6113218 | 2.1510494 | 0 | 0.024344277 | 0.177 |
| GOBP_POSITIVE_REGULATION_OF_REGULATORY_T_CELL_DIFFERENTIATION | 0.8483467 | 2.150091 | 0 | 0.023074182 | 0.179 |
| GOBP_REGULATION_OF_B_CELL_PROLIFERATION | 0.632321 | 2.1386626 | 0.00203252 | 0.02465494 | 0.198 |
| GOBP_REGULATION_OF_LEUKOCYTE_PROLIFERATION | 0.53174436 | 2.1378415 | 0.01039501 | 0.023298193 | 0.198 |
| GOBP_REGULATION_OF_RESPIRATORY_BURST | 0.75411564 | 2.131515 | 0 | 0.023598153 | 0.203 |
| GOBP_ADAPTIVE_IMMUNE_RESPONSE_BASED_ON_SOMATIC_RECOMBINATION_OF_IMMUNE_RECEPTORS_BUILT_FROM_IMMUNOGLOBULIN_SUPERFAMILY_DOMAINS | 0.6043383 | 2.130831 | 0 | 0.022486825 | 0.203 |
| GOBP_INTERLEUKIN_4_PRODUCTION | 0.6653829 | 2.128613 | 0 | 0.021934122 | 0.206 |
| GOBP_POSITIVE_REGULATION_OF_B_CELL_PROLIFERATION | 0.62951934 | 2.1286118 | 0.002028398 | 0.02088964 | 0.206 |
| GOBP_RESPONSE_TO_INTERFERON_BETA | 0.6881828 | 2.1273236 | 0.00610998 | 0.020126851 | 0.206 |
| GOBP_HUMORAL_IMMUNE_RESPONSE | 0.59177977 | 2.1220748 | 0 | 0.020667855 | 0.211 |
| GOBP_NEGATIVE_REGULATION_OF_INTERLEUKIN_1_BETA_PRODUCTION | 0.58728707 | 2.1219304 | 0 | 0.019833794 | 0.211 |
| GOBP_DEFENSE_RESPONSE_TO_BACTERIUM | 0.5154629 | 2.1142998 | 0 | 0.020861637 | 0.217 |
| GOBP_INTERLEUKIN_10_PRODUCTION | 0.5949007 | 2.1114097 | 0.005988024 | 0.020809688 | 0.223 |
| GOBP_CELLULAR_RESPONSE_TO_ZINC_ION | 0.71878386 | 2.1113696 | 0 | 0.020038959 | 0.223 |
| GOBP_RESPONSE_TO_PROTOZOAN | 0.7498475 | 2.1086307 | 0.003944773 | 0.020031929 | 0.226 |
| GOBP_POSITIVE_REGULATION_OF_LEUKOCYTE_CELL_CELL_ADHESION | 0.55284125 | 2.1067932 | 0.014492754 | 0.019801607 | 0.229 |
| GOBP_POSITIVE_REGULATION_OF_CELL_ACTIVATION | 0.5417746 | 2.100492 | 0.00203252 | 0.020685103 | 0.24 |
| GOBP_TUMOR_NECROSIS_FACTOR_MEDIATED_SIGNALING_PATHWAY | 0.5332871 | 2.0997903 | 0.008528785 | 0.02020483 | 0.243 |
| GOBP_NEGATIVE_REGULATION_OF_ADAPTIVE_IMMUNE_RESPONSE | 0.59007025 | 2.098992 | 0.001992032 | 0.019666268 | 0.243 |
| GOBP_HUMORAL_IMMUNE_RESPONSE_MEDIATED_BY_CIRCULATING_IMMUNOGLOBULIN | 0.7752797 | 2.096101 | 0.002 | 0.01980447 | 0.25 |
| GOBP_LEUKOCYTE_PROLIFERATION | 0.49901888 | 2.0946543 | 0.008316008 | 0.01957226 | 0.251 |
| GOBP_T_CELL_PROLIFERATION | 0.5373269 | 2.0923772 | 0.008602151 | 0.019521892 | 0.252 |
| GOBP_B_CELL_MEDIATED_IMMUNITY | 0.6716124 | 2.0914767 | 0.002004008 | 0.019096566 | 0.252 |
| GOBP_INTERLEUKIN_1_PRODUCTION | 0.50672823 | 2.0898693 | 0.001890359 | 0.018873416 | 0.252 |
| GOBP_REGULATORY_T_CELL_DIFFERENTIATION | 0.74389046 | 2.0858676 | 0.002008032 | 0.019152494 | 0.255 |
| GOBP_NEGATIVE_REGULATION_OF_IMMUNE_RESPONSE | 0.529102 | 2.084834 | 0.004056795 | 0.018887836 | 0.257 |
| GOBP_B_CELL_PROLIFERATION | 0.54341036 | 2.0837672 | 0.005940594 | 0.018583426 | 0.258 |
| GOBP_REGULATION_OF_ANTIGEN_PROCESSING_AND_PRESENTATION | 0.7999962 | 2.0731907 | 0.00407332 | 0.020606786 | 0.272 |
| GOBP_NEGATIVE_REGULATION_OF_NATURAL_KILLER_CELL_MEDIATED_IMMUNITY | 0.78666556 | 2.0727715 | 0.005825243 | 0.02017448 | 0.273 |
| GOBP_RESPONSE_TO_ZINC_ION | 0.543686 | 2.0725188 | 0 | 0.019717347 | 0.273 |
| GOBP_INTERLEUKIN_1_BETA_PRODUCTION | 0.5180494 | 2.069461 | 0.003795066 | 0.019989753 | 0.277 |
| GOBP_DETECTION_OF_BIOTIC_STIMULUS | 0.6366429 | 2.0660722 | 0.003898636 | 0.0200634 | 0.283 |
| GOBP_ANTIGEN_PROCESSING_AND_PRESENTATION_OF_EXOGENOUS_PEPTIDE_ANTIGEN_VIA_MHC_CLASS_I | 0.65994376 | 2.0597997 | 0.004132231 | 0.02101463 | 0.295 |
| GOBP_NEGATIVE_REGULATION_OF_INNATE_IMMUNE_RESPONSE | 0.5859517 | 2.0566928 | 0.004016064 | 0.021288589 | 0.302 |
| GOBP_NECROTIC_CELL_DEATH | 0.5551914 | 2.0485528 | 0 | 0.022803504 | 0.321 |
| GOBP_POSITIVE_REGULATION_OF_ALPHA_BETA_T_CELL_ACTIVATION | 0.61045796 | 2.0467122 | 0.012631579 | 0.022780254 | 0.329 |
| GOBP_DEFENSE_RESPONSE_TO_GRAM_POSITIVE_BACTERIUM | 0.5087589 | 2.0424426 | 0 | 0.023463048 | 0.336 |
| GOBP_ACUTE_INFLAMMATORY_RESPONSE | 0.5727594 | 2.042216 | 0 | 0.023075065 | 0.337 |
| GOBP_DETOXIFICATION_OF_INORGANIC_COMPOUND | 0.79696286 | 2.0415719 | 0.001956947 | 0.022809152 | 0.339 |
| GOBP_T_HELPER_1_TYPE_IMMUNE_RESPONSE | 0.60633785 | 2.0346391 | 0.004081633 | 0.024116956 | 0.351 |
| GOBP_NEGATIVE_REGULATION_OF_CELL_KILLING | 0.68153936 | 2.0339878 | 0.007692308 | 0.023795323 | 0.351 |
| GOBP_INTERFERON_GAMMA_MEDIATED_SIGNALING_PATHWAY | 0.6248142 | 2.0336223 | 0.012024048 | 0.023464048 | 0.352 |
| GOBP_NEGATIVE_REGULATION_OF_LEUKOCYTE_MEDIATED_CYTOTOXICITY | 0.7404601 | 2.03289 | 0.007766991 | 0.02323438 | 0.352 |
| GOBP_REGULATION_OF_T_CELL_ACTIVATION | 0.5086695 | 2.030175 | 0.017094018 | 0.023437731 | 0.357 |
| GOBP_RESPONSE_TO_COPPER_ION | 0.5828807 | 2.0265894 | 0.001953125 | 0.023902925 | 0.36 |
| GOBP_T_CELL_MEDIATED_IMMUNITY | 0.5592482 | 2.023684 | 0.010224949 | 0.02416178 | 0.365 |
| GOBP_REGULATION_OF_B_CELL_ACTIVATION | 0.60545665 | 2.0231106 | 0.008032128 | 0.023900082 | 0.367 |
| GOBP_T_CELL_ACTIVATION_INVOLVED_IN_IMMUNE_RESPONSE | 0.51910734 | 2.0222352 | 0.008230452 | 0.023696102 | 0.367 |
| GOBP_POSITIVE_REGULATION_OF_CELL_CELL_ADHESION | 0.50553316 | 2.0210054 | 0.014344262 | 0.023695484 | 0.37 |
| GOBP_REGULATION_OF_T_CELL_MEDIATED_IMMUNITY | 0.5692022 | 2.0162883 | 0.012024048 | 0.024460237 | 0.375 |
| GOBP_REGULATION_OF_LYMPHOCYTE_ACTIVATION | 0.50337374 | 2.0159495 | 0.006160164 | 0.024132147 | 0.375 |
| GOBP_STRESS_RESPONSE_TO_METAL_ION | 0.77056336 | 2.0152948 | 0.005976096 | 0.023911988 | 0.376 |
| GOBP_NEGATIVE_REGULATION_OF_LYMPHOCYTE_ACTIVATION | 0.5031617 | 2.0142035 | 0.012 | 0.023800908 | 0.378 |
| GOBP_REGULATION_OF_T_HELPER_1_TYPE_IMMUNE_RESPONSE | 0.63687986 | 2.0133152 | 0.001976285 | 0.023688551 | 0.383 |
| GOBP_POSITIVE_REGULATION_OF_B_CELL_ACTIVATION | 0.628668 | 2.0069177 | 0.006036217 | 0.024885137 | 0.399 |
| GOBP_INTERLEUKIN_13_PRODUCTION | 0.60295624 | 2.0067334 | 0 | 0.024537288 | 0.399 |
| GOBP_REGULATION_OF_ALPHA_BETA_T_CELL_ACTIVATION | 0.5612391 | 2.0060985 | 0.018947368 | 0.024362085 | 0.4 |
| GOBP_LYMPHOCYTE_COSTIMULATION | 0.6161453 | 2.0055833 | 0.024691358 | 0.024134094 | 0.402 |
| GOBP_REGULATION_OF_KILLING_OF_CELLS_OF_OTHER_ORGANISM | 0.6956978 | 2.0037332 | 0 | 0.024229392 | 0.406 |
| GOBP_REGULATION_OF_NECROTIC_CELL_DEATH | 0.5570486 | 1.9955705 | 0 | 0.025944412 | 0.415 |
| GOBP_LEUKOCYTE_MIGRATION_INVOLVED_IN_INFLAMMATORY_RESPONSE | 0.7184338 | 1.9953483 | 0.003752345 | 0.025643952 | 0.415 |
| GOBP_LEUKOCYTE_CELL_CELL_ADHESION | 0.48451096 | 1.989928 | 0.018442623 | 0.026748562 | 0.43 |
| GOBP_ALPHA_BETA_T_CELL_ACTIVATION | 0.5401153 | 1.9897271 | 0.016877636 | 0.02643369 | 0.431 |
| GOBP_CELLULAR_DEFENSE_RESPONSE | 0.61666924 | 1.9847047 | 0.01814516 | 0.027334778 | 0.441 |
| GOBP_T_HELPER_2_CELL_DIFFERENTIATION | 0.69152975 | 1.9807608 | 0.006122449 | 0.028050805 | 0.449 |
| GOBP_CELL_KILLING | 0.5035049 | 1.9773856 | 0.005964215 | 0.028623082 | 0.453 |
| GOBP_COMPLEMENT_ACTIVATION | 0.6927403 | 1.975196 | 0.005905512 | 0.028834358 | 0.46 |
| GOBP_ANTIGEN_RECEPTOR_MEDIATED_SIGNALING_PATHWAY | 0.5434001 | 1.971898 | 0.016359918 | 0.029341606 | 0.468 |
| GOBP_ACUTE_PHASE_RESPONSE | 0.65910953 | 1.9707901 | 0.003690037 | 0.029272815 | 0.472 |
| GOBP_REGULATION_OF_ADAPTIVE_IMMUNE_RESPONSE | 0.5029147 | 1.9694884 | 0.012145749 | 0.029258806 | 0.477 |
| GOBP_POSITIVE_REGULATION_OF_CD4_POSITIVE_ALPHA_BETA_T_CELL_ACTIVATION | 0.60942256 | 1.9642156 | 0.016161617 | 0.030358722 | 0.485 |
| GOBP_NEGATIVE_REGULATION_OF_T_CELL_MEDIATED_IMMUNITY | 0.6754208 | 1.9598187 | 0.009689922 | 0.031222964 | 0.498 |
| GOBP_NEGATIVE_REGULATION_OF_INTERLEUKIN_1_PRODUCTION | 0.5159268 | 1.9587898 | 0.00189394 | 0.031112641 | 0.498 |
| GOBP_LEUKOCYTE_CHEMOTAXIS | 0.48118788 | 1.9562184 | 0.01004016 | 0.031481117 | 0.509 |
| GOBP_REGULATION_OF_TOLL_LIKE_RECEPTOR_SIGNALING_PATHWAY | 0.5422257 | 1.9555074 | 0.002016129 | 0.03136272 | 0.512 |
| GOBP_T_CELL_ACTIVATION | 0.4724612 | 1.9553026 | 0.02096436 | 0.031122092 | 0.514 |
| GOBP_REGULATION_OF_INFLAMMATORY_RESPONSE_TO_ANTIGENIC_STIMULUS | 0.64011157 | 1.9541428 | 0.010438413 | 0.031080553 | 0.514 |
| GOBP_LEUKOCYTE_MEDIATED_CYTOTOXICITY | 0.537981 | 1.953425 | 0.018036073 | 0.030932492 | 0.515 |
| GOBP_TOLL_LIKE_RECEPTOR_SIGNALING_PATHWAY | 0.50902444 | 1.953283 | 0.00998004 | 0.030616159 | 0.515 |
| GOBP_POSITIVE_REGULATION_OF_CYTOKINE_PRODUCTION | 0.45913658 | 1.953222 | 0.00617284 | 0.030312503 | 0.515 |
| GOBP_REGULATION_OF_HUMORAL_IMMUNE_RESPONSE | 0.66545314 | 1.9512248 | 0.004 | 0.030545516 | 0.523 |
| GOBP_REGULATION_OF_CELL_KILLING | 0.54152626 | 1.94889 | 0.023809524 | 0.03085105 | 0.53 |
| GOBP_NEGATIVE_REGULATION_OF_INTERLEUKIN_12_PRODUCTION | 0.7720498 | 1.9475309 | 0.007827789 | 0.030971635 | 0.532 |
| GOBP_REGULATION_OF_CD4_POSITIVE_ALPHA_BETA_T_CELL_ACTIVATION | 0.5473266 | 1.9474118 | 0.016129032 | 0.030676395 | 0.532 |
| GOBP_ACUTE_INFLAMMATORY_RESPONSE_TO_ANTIGENIC_STIMULUS | 0.6576266 | 1.9464915 | 0 | 0.030621162 | 0.532 |
| GOBP_NEGATIVE_REGULATION_OF_LEUKOCYTE_PROLIFERATION | 0.52205145 | 1.9442687 | 0.026584867 | 0.030934483 | 0.538 |
