# Supplementary material for: Identification of a Methylation-Regulating Genes Prognostic Signature to Predict the Prognosis and Aid Immunotherapy of Clear Cell Renal Cell Carcinoma
Source: Front Cell Dev Biol. 2022 Mar 2;10:832803. doi: 10.3389/fcell.2022.832803 (PMC8924039; doi:10.3389/fcell.2022.832803)
Supplement: Supplementary file 7 [file Table2.DOC]

**S Table 2.**Thirteen hub genes were identified as candidate genes between DEGs1 and DEGs2.

| BHMT | MAPT-AS1 | PNMT |
| --- | --- | --- |
| CYP1A1 | MECOM | PRDM16 |
| FOS | NOP2 | SETMAR |
| NSUN6 | TET2 | TDRD5 |
| WT1 |  |  |
